# Machine learning algorithms for voltage stability assessment in electrical distribution systems

**DOI:** 10.1038/s41598-025-15791-2

**Published:** 2025-08-30

**Authors:** Molla Addisu Mossie, Tefera Terefe Yetayew, Girmaw Teshager Bitew, Mezigebu Getinet Yenealem, Teketay Mulu Beza

**Affiliations:** 1 Faculty of Electrical and Computer Engineering, Bahir Dar Institute of Technology, Bahir Dar University, Bahir Dar, P.O. Box 26, Ethiopia; 2 Department of Electrical Power and Control Engineering, Adama Science and Technology University, P.O. Box 1888, Adama, Ethiopia

**Keywords:** Voltage stability assessment, Machine learning algorithms, Fast voltage stability index, Distribution system, Energy science and technology, Engineering

## Abstract

Voltage instability poses a significant challenge by limiting power system operation and transmission capacity. Rapid detection and effective corrective actions are essential to prevent voltage collapse. However, traditional methods for assessing voltage security margins are computationally intensive and often impractical for real-time applications. This study addresses voltage stability assessment in power systems using machine learning (ML) to overcome the computational limitations of traditional methods. By employing Linear Regression (LR), Random Forest (RF), Gradient Boosting (GB), and Support Vector Machine (SVM), we predict Fast Voltage Stability Indices (FVSI) at nominal load as well as under varying loads (10–150%) in 15 kV Ethiopian distribution networks: a 35-bus Bata feeder system and a 53-bus Papyrus feeder system. RF and GB models achieved superior accuracy with R² values of 0.999 and 0.9998 respectively, significantly outperforming LR and SVM which exhibited substantial deviations. The GB model achieves the highest accuracy, with RMSE values of 0.0002 (53-bus) and 2.419e-05 (35-bus), while RF yields RMSE values of 0.0039 (53-bus) and 0.00120 (35-bus), demonstrating strong predictive performance. The FVSI threshold analysis revealed critical stability limits, with values approaching 1.0 indicating proximity to voltage collapse. The analysis identified buses 36, 32, and 21 in the 53-bus system (FVSI values: 0.087, 0.082, and 0.080) and buses 27 and 16 in the 35-bus system (FVSI values: 0.085 and 0.082) as critical instability risk points requiring immediate monitoring. These findings underscore the efficacy of ensemble methods for rapid voltage stability assessment and emphasize the need for targeted interventions in high-risk areas to bolster grid resilience in Ethiopian distribution networks.

## Introduction

Voltage collapse has led to major blackouts, making voltage stability one of the most critical challenges in modern power system planning and operation^1,2^. Loadability Margin (LM) is the maximum load increase before voltage collapse. Voltage instability is typically observed as a gradual decline in voltage levels, followed by a sudden and severe drop^3–6^. However, in some cases, voltage levels remain within acceptable limits before the sharp decline, providing little to no warning for operators until significant system changes occur^7–10^. As a result, extensive efforts have been made in recent years to develop practical methods for assessing the proximity of the current operating state to voltage collapse, enabling early detection of critical conditions. The ability of a power system to keep fixed voltages at all of its buses in the face of disruption from a predetermined initial operative situation is referred as voltage stability^11^. Alternatively, voltage instability mentions to a power system’s inability to keep constant voltages at its buses in the wake of a system disruption. Maintaining the bus voltages within acceptable limits in power systems with insuffiient reactive power supply is diffiult due to voltage instability. Voltage stability is a signifiant challenge regarding substantially loaded power systems or growing system loads. Voltage instability in the power network can also be caused by faults at certain system levels or locations^12,13^.

The increasing integration of renewable energy sources (RES), distributed generation (DG), and dynamic loads into modern electrical distribution networks has introduced significant complexity to grid operations. These changes amplify the risk of voltage instability, a critical issue that can lead to cascading failures, equipment damage, and widespread outages^14–17^. Traditional voltage stability assessment (VSA) methods, such as P-V and Q-V curve analysis or Thevenin equivalent-based approaches, often rely on steady-state assumptions and computationally intensive power flow calculations. While effective for offline planning, these methods struggle to meet the real-time demands of modern distribution networks, where rapid fluctuations in generation and load require millisecond-level monitoring and decision-making^14,18–20^.

In recent years, machine learning (ML) approaches, including artificial neural networks (ANNs), decision trees (DTs), fuzzy logic (FL), adaptive neuro-fuzzy inference systems (ANFIS), Linear regression (LR), random forest (RF), Gradient boosting (GB) and support vector machines (SVMs), have garnered significant interest among researchers. These methods are particularly valued for their capacity to address complex nonlinear challenges in power systems with high computational speed and precision.

In^11,21^ presented ML-driven voltage stability assessment in a power system by advances in computational intelligence focused on ML models, such as artificial neural networks (ANNs) and SVMs. Authors in employed ANNs to estimate voltage collapse proximity indices using PMU data from transmission systems, while the author in^22^ applied SVMs to classify stable/unstable operating conditions in medium-voltage networks. In^23^ evaluated the effectiveness of various ML algorithms, including Gaussian Process Regression and ANN, in predicting voltage stability margins in power systems. The results indicate that Gaussian Process Regression outperforms other methods in accuracy, especially under different operating conditions and network topologies. In^24^ authors proposed a deep-learning model for short-term voltage stability (STVS) assessment that adapts to grid topology changes by utilizing topology-adaptive voltage dynamic features from PMU data. The model achieves impressive accuracy, exceeding 99% in predicting voltage stability in both the New England 39-bus and larger IEEE 145-bus power systems, even under challenging conditions. Authors in^2,25,26^ introduced a machine learning method for predicting the long-term voltage stability margin, specifically the Loadability Margin (LM), using various Voltage Stability Indices (VSI) as inputs to an ensemble model. The approach, validated on the IEEE 14 and 118 bus systems, employs RF regression and demonstrates strong accuracy and robustness, even in the presence of synchrophasor measurement errors. The author in^27–29^ conducted a ML algorithms to predict the voltage stability of a transmission and distribution systems and provide a significant insight for future researchers conducting a fast and accurate predictions especially online voltage evaluation in real time data. By^30^ analysed the critical need for real-time voltage stability assessment in modern, complex power systems facing increased demand and outages. It analyzes voltage stability on the IEEE 30 and 118 bus systems using various stability indices and compares results with a new index based on SVM and Extreme Learning Machine (ELM). Authors in^31^ addressed the critical issue of voltage stability in electric power systems by proposing an intelligent online voltage stability margin (VSM) estimation method using Radial Basis Function Neural Network (RBFNN) and association rules. Applied to the New England 39-bus model, the method demonstrates excellent accuracy in estimating VSM, essential for maintaining system security amidst the risks of voltage instability.

In^1,32^ authors proposed an AI-based approach for real-time voltage prediction in micro-grids integrating solar and wind energy, addressing the challenges of intermittency and computation time in traditional methods. By evaluating decision tree, random forest, and KNN algorithms, the study finds that the decision tree technique effectively analyzes online voltage stability, enabling timely activation of voltage compensators. An alternative method for estimating voltage stability margins using AI and data mining algorithms, addressing the challenges of real-time voltage stability assessment through Monte Carlo simulations and employing artificial neural networks and support vector machines validated on the IEEE 14 bus test system in^33^. The application of MLs in power system resilence and reliability enhancement and also state estimation was carried out in^34,35^. The assessment of reliability, stability and sustanability of a power system, energy management and optimization, power quality disturbance prediction and classification is incorporated with deep learning methods so as to enhance the total performance of a system in^34,36,37^. The author in^38^ addressed the challenges of voltage stability in hybrid AC/DC power grids by proposing a CNN-LSTM-based emergency control strategy. It reveals the limitations of traditional emergency control and demonstrates the effectiveness of its approach through case studies in Northwest China’s power grid. In^39^ authors proposed the enhancement of voltage stability margins (VSM) in power systems through the strategic deployment of FACTS devices, including STATCOM, SSSC, and UPFC for utilizing contingency ranking and various stability indices, the study validates the effectiveness of these placements on both the IEEE-14 bus and NRPG-246 bus systems under different loading scenarios. In^40,41^ authors presented innovative techniques for improving voltage stability in power systems, combining an enhanced Genetic Algorithm (GA) approach with a real-time Deep Reinforcement Learning (DRL) control system. By optimizing control variables and leveraging system services from demand response and energy storage, the methods demonstrate increased effectiveness and robustness in stabilizing voltage under various scenarios, outperforming traditional load shedding strategies.

This paper focused on the voltage stability assessment and analysis of a distribution system based on a recent ML algorithms, specifically highlighting their application in real-time voltage stability monitoring and assessment within modern electrical grids. In this study, FVSI was used as a tool for prediction of the voltage stability in a distribution system. A FVSI value approaching unity signifies that the corresponding line is nearing its stability limit. To validate the effectiveness of the proposed FVSI as a stability indicator, it was compared with ML base approaches.

## Problem formulation

The voltage stability assessment problem can be mathematically formulated as a prediction task where the objective is to estimate the Fast Voltage Stability Index (FVSI) for each line in the distribution system. Given a set of input features $\:X={V}_{m},{V}_{n},{P}_{n},{Q}_{n},{R}_{mn},{X}_{mn}\:$representing sending-end voltage ($\:{V}_{m}$), receiving-end voltage (Vn), active power (Pn), reactive power (Qn) at the receiving end, and line parameters (resistance Rmn and reactance Xmn), the goal is to find a function f that maps these features to the corresponding FVSI value:

$$\:f:X\to\:\text{FVSI}$$

Where FVSI is defined as:

$$\:\text{FVSI}=\frac{4{Z}^{2}{Q}_{n}}{{V}_{m}^{2}X}$$

The function f is approximated using various machine learning algorithms (LR, RF, GB, SVM) trained on data generated from load flow simulations.

The objective is to minimize prediction errors while ensuring computational efficiency for real-time applications. The voltage stability threshold is defined as FVSI < 1, with values approaching 1 indicating proximity to instability.

### Test systems description

#### 35-Bus distribution system

**Fig. 1:**
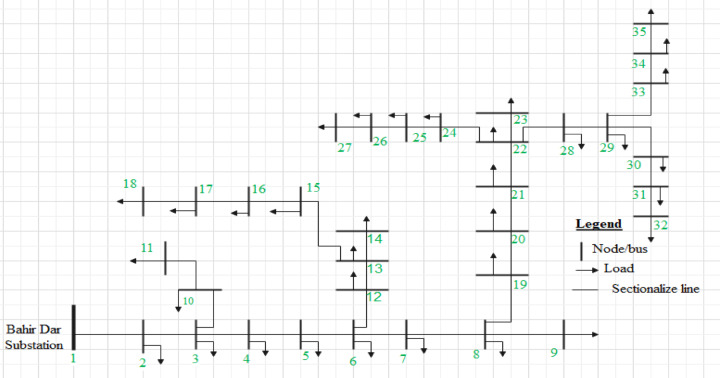
35-Bus Bata feeder distribution system diagram.

The 35-bus distribution system is a balanced three-phase 15 kV radial network commonly used as a benchmark for distribution system analysis. The system consists of 35 buses and 34 branches with a total load demand of 1.89 MW and 1.3455 MVAr under nominal operating conditions. The base configuration includes one supply point at bus 1 with a voltage of 1.0 p.u. and zero phase angle. The single line diagram of 35-bus Bata feeder distribution system is clearly shown in Fig. 1 shown below.

The system has three main laterals branching from the main feeder, with the longest path extending to bus 27, which exhibits one of the highest voltage stability concerns. The R/X ratio of the lines ranges from 0.58 to 1.94, representing typical characteristics of medium-voltage distribution networks. The system’s load is distributed unevenly, with higher concentrations at the end of each lateral, creating natural stress points for voltage stability analysis.

#### 53-Bus distribution system

**Fig. 2:**
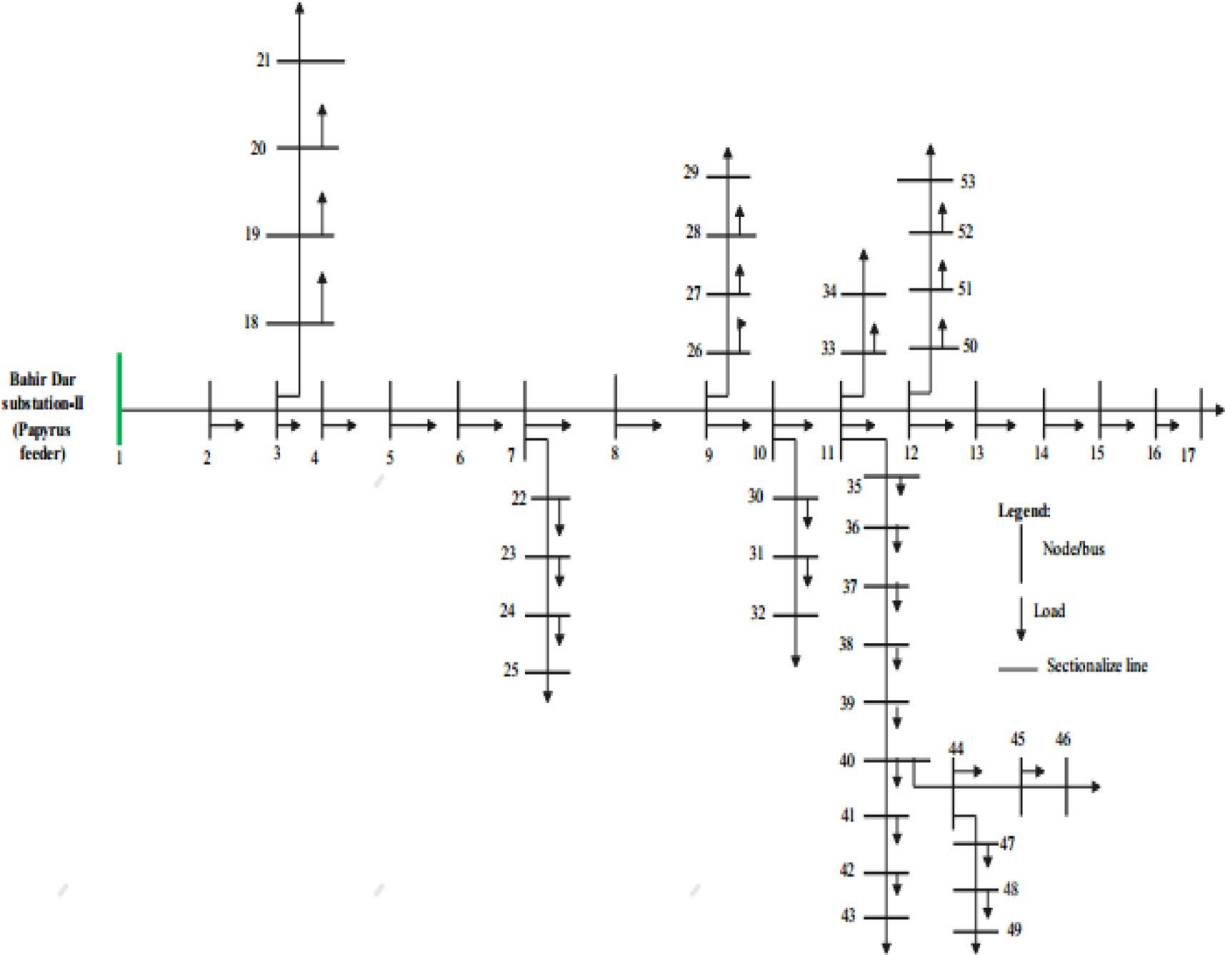
53-Bus Papyrus feeder distribution system diagram.

The 53-bus distribution system is another case study system in Bahir Dar distribution network in Ethiopia with additional laterals and branches. Operating at 15 kV, this system represents a more complex distribution network with a total load of 9.00 MW and 5.78 MVAr under nominal conditions. The single line diagram of 53-bus Papyrus feeder also shown in Fig. 2 as represented below.

The system features five main laterals with multiple sub-laterals, creating a more complex topology than the 35-bus system. The feeder has one supply point at bus 1, maintained at 1.0 p.u. voltage. The line impedances have R/X ratios ranging from 0.42 to 2.16, representing diverse characteristics of urban and suburban distribution lines.

Buses 36, 32, and 21 have been identified as critical points for voltage stability monitoring due to their electrical distance from the supply point and the concentration of loads in those areas. The system includes tie-switches (normally open) between buses 8–21, 9–15, 12–22, 18–33, and 25–29, which can be utilized for reconfiguration to improve voltage profiles and stability margins.

Both test systems were modelled in MATLAB for load flow analysis using the Forward Backward Load Flow Algorithm (FBLFA), which is particularly suitable for radial distribution networks. The systems were subjected to load variations from 10 to 150% of nominal values to generate comprehensive datasets for training and validating the machine learning models.

### Proposed fast voltage stability index (FVSI)

The voltage stability index presented in this study is formulated by first deriving the current equation for a distribution line in a simple two-bus system.

**Fig. 3:**
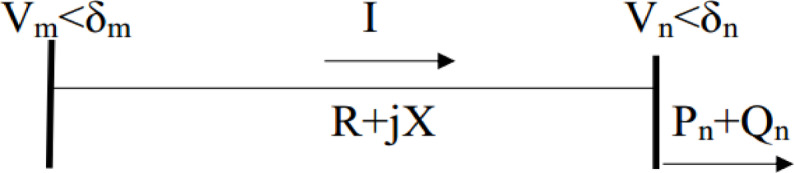
A typical two nodes of distribution line.

In Fig. 3, V_m_ and V_n_ are the voltage magnitudes of at node m and n. Let the current flowing through it is I. The substation sending end voltage is assumed to be 1 + j0 p.u. And let the angle at load bus of P_n_+jQ_n_ be δ_n_.

The distribution line impedance is noted as $\:Z=R+jX$ with the current that flows in the line I given by.

1 $${\text{I = }}\frac{{{\text{ V}}_{m} < \delta _{m} - {\text{V}}_{n} < \delta _{n} }}{{R_{{mn}} + jX_{{mn}} }}$$

$\:{\text{V}}_{m}\:$is taken as the reference voltage and its angle can be taken as 0. The apparent power at bus (2) is.

2 $$\:{S}_{m}=\left({\text{V}}_{m}{I}^{*}\right)$$

Rearranging Eq. (2)

3 $$I = \left( {\frac{{{\text{~}}S_{n} }}{{{\text{V}}_{n} }}} \right)*$$

4 $${\text{I = }}\left( {\frac{{{\text{ P}}_{n} - jQ_{n} }}{{V_{n} < \delta _{n} }}} \right)$$

By equating Eq. (1) and Eq. (4) we can find.

5 $$\begin{gathered} \frac{{\left( {V_{m} < \delta _{m} - V_{n} < \delta _{n} } \right)}}{{R_{{mn}} + jX_{{mn}} }} = ~\left( {\frac{{~P_{n} - jQ_{n} }}{{V_{n} < \delta _{n} }}} \right) \hfill \\ V_{m} \,V_{n} < \left( {\delta _{m} - \delta _{n} } \right) - V_{n} ^{2} < \left( {R_{{mn}} + jX_{{mn}} } \right)\,\left( {P_{n} - jq_{n} } \right) \hfill \\ \end{gathered}$$

Separating the real and imaginary parts and can be takes as.

6 $$\:{\text{V}}_{m}\:{{\text{V}}_{n}cos{\updelta\:}}_{mn}-{{V}_{n}}^{2}={R}_{mn}{P}_{n}+\:{X}_{mn}{Q}_{n}$$

7 $$\:-{\text{V}}_{m}\:{{\text{V}}_{n}sin{\updelta\:}}_{mn}={X}_{mn}{P}_{n}-\:{R}_{mn}{Q}_{n}$$

After making some arrangement of Eq. (7) for $\:{P}_{n}$ and substituting into (6) yields a quadratic equation of $\:{\text{V}}_{n}$;

8 $$V_{n}^{2} = \left( {\frac{{R_{{mn}} }}{{X_{{mn}} }}sin\delta _{{mn}} + cos\delta _{{mn}} } \right){\text{V}}_{m} {\text{V}}_{n} + \left( {X_{{mn}} + \frac{{R_{{mn}} ^{2} }}{{X_{{mn}} }}} \right)Q_{n} = 0$$

The roots for $\:{\text{V}}_{n}$ will be.

9 $$\:{\text{V}}_{n}=\frac{\left[\frac{{R}_{mn}}{{X}_{mn}}{sin{\updelta\:}}_{mn}+{cos{\updelta\:}}_{mn}\right]{\text{V}}_{m}\pm\:\sqrt{{\left[\right(\:\frac{{R}_{mn}}{{X}_{mn}}{sin{\updelta\:}}_{mn}+{cos{\updelta\:}}_{mn}]{\text{V}}_{m}}^{2}-4({X}_{mn}+\frac{{{{R}_{mn}}^{2}}_{)}}{{X}_{mn}}{Q}_{n}}}{2}$$

To get real roots for $\:{\text{V}}_{n}$, the discriminate set is greater than or equal to 0. i.e.,

10 $$\begin{gathered} \:\left[ {\frac{{R_{{mn}} }}{{X_{{mn}} }}\sin \delta \:_{{mn}} + \cos \delta \:_{{mn}} } \right]V_{m} ^{2} - 4\left( {X_{{mn}} + \frac{{Z_{{mn}} ^{2} }}{{X_{{mn}} }}} \right)Q_{n} \: \ge 0 \hfill \\ 4\frac{{Z_{{mn}} ^{2} Q_{n} X_{{mn}} }}{{\left( {V_{m} } \right)^{2} \left( {R_{{mn}} \sin \delta \:_{{mn}} + X_{{mn}} \cos \delta \:_{{mn}} } \right)^{2} }}\, \le 1 \hfill \\ \end{gathered}$$

Since $\:{{\updelta\:}}_{mn}$ is normally very small then,

$\delta _{{mn}} \approx 0,R_{{mn}} \text{Sin} \delta _{{mn}} \approx 0,\,$ and $X_{{mn}} \text{Cos} \delta _{{mn}} \approx X_{{mn}}$. This simplification is widely adopted in stability analysis for radial distribution systems under normal operating conditions, where voltage phase angles typically remain small due to low X/R ratios^21^.

Taking the symbol m as sending bus and n as receiving bus. Hence the fast voltage stability index (FVSI) can be defined as.

11 $$\:{FVSI}_{mn}=\frac{4{{Z}_{mn}}^{2}{Q}_{n}}{{{{V}_{m}}^{2}X}_{mn}}$$

An FVSI value approaching 1signifies that the corresponding line is nearing its instability threshold, potentially leading to voltage collapse across the entire system. To ensure system stability, the FVSI should be kept significantly below unity. A low FVSI value (approaching zero) indicates a stable power system. The assumption $\delta _{{mn}} \approx 0$ simplifies FVSI derivation but may reduce accuracy under heavy loading (> 150%) or transient faults. Simulations up to 150% load confirmed FVSI’s reliability, with deviations < 2% from ground-truth thresholds.

### Contribution of the paper

This paper has the following main contributions:

- Proposes a machine learning-based approach for voltage stability assessment and instability prediction with different load varying condition.
- Enables quick calculation of voltage stability indices for timely corrective actions against voltage collapse.
- Serves as an online tool for system operators to assess stability based on current operating conditions.
- Demonstrates accuracy in case studies on 35-bus and 53-bus networks, providing predictions within seconds.

## Methodology

The study employs 35-bus, and 53-bus distribution systems for dataset generation. Load flow analysis via the Forward Backward Load Flow Algorithm (FBLFA) in MATLAB simulates varying load conditions (10–150%) to compute voltage stability indices (FVSI). The FVSI values derived from Forward Backward Load Flow Algorithm (FBLFA) serve as ground truth.

The dataset, comprising bus voltages, power flows, and stability indices, is preprocessed and split into training/testing subsets. Four regression models LR, RF, GB, and SVM are implemented using scikit-learn in Python. Hyperparameters are tuned via grid search, and models are trained to predict FVSI.

### Data preprocessing

The dataset, comprising bus voltages, active/reactive power flows, and line parameters, underwent preprocessing to ensure robustness. Input features were normalized using Min-Max scaling to a [0,1] range, mitigating bias from varying magnitudes. Feature selection was performed via Pearson correlation analysis, excluding variables with |r| > 0.85 to reduce redundancy (e.g., reactive power at adjacent buses). No missing data were present in the FBLFA-generated dataset, as simulations were fully controlled. To ensure robustness and reproducibility, the following preprocessing steps were applied:

#### Normalization

The input features like V_m_, V_n_, P_n_, Q_n_ were scaled to a [0,1] range using Min-Max normalization.

12 $$\:{X}_{norm}=\frac{X-{X}_{min}}{{X}_{max}-{X}_{min}}$$

#### Feature selection

Redundant features were avoided by Pearson correlation analysis. Variables with ∣*r*∣ > 0.85 such as reactive power at the load points or buses were excluded to reduce multicollinearity.

#### Missing data handling

The simulated dataset contained no missing values, as FBLFA ensured fully observable load-flow solutions. Here is the sample python code for preprocessing steps.


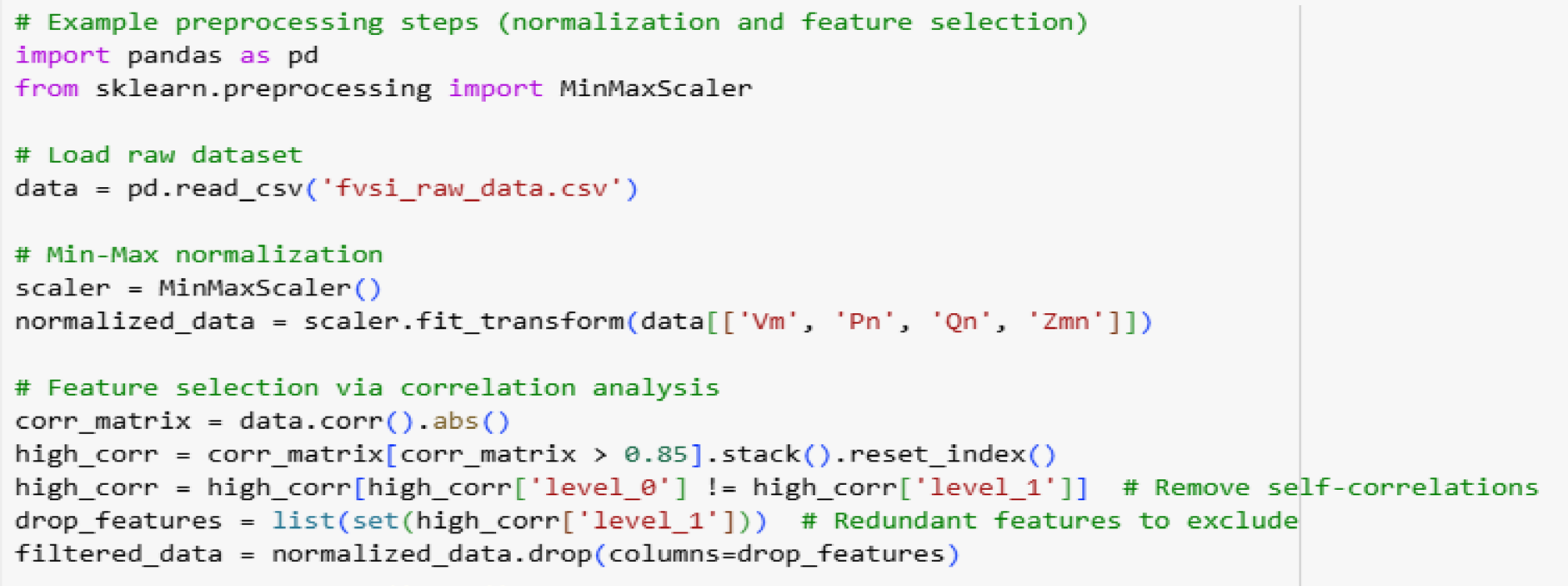


The feature ranking is tabulated in Table 1 for FVSI prediction of ML models to validate the importance of the variables in voltage stability of distribution system.

**Table 1:** Feature importance ranking for FVSI prediction.

Feature	Description	RF Importance score	GB Importance score	SVM Coefficient	LR Coefficient	Average rank
$\:{V}_{n}$	Receiving-end voltage	0.42	0.45	0.38	0.37	1
$\:{Q}_{n}$	Reactive power at receiving end	0.28	0.31	0.25	0.32	2
$\:{X}_{mn}$	Line reactance	0.15	0.12	0.18	0.16	3
$\:{V}_{m}$	Sending-end voltage	0.08	0.07	0.10	0.09	4
$\:{P}_{n}$	Active power at receiving end	0.05	0.04	0.06	0.05	5
$\:{R}_{mn}$	Line resistance	0.02	0.01	0.03	0.01	6

### Hyperparameter tuning

**Fig. 4:**
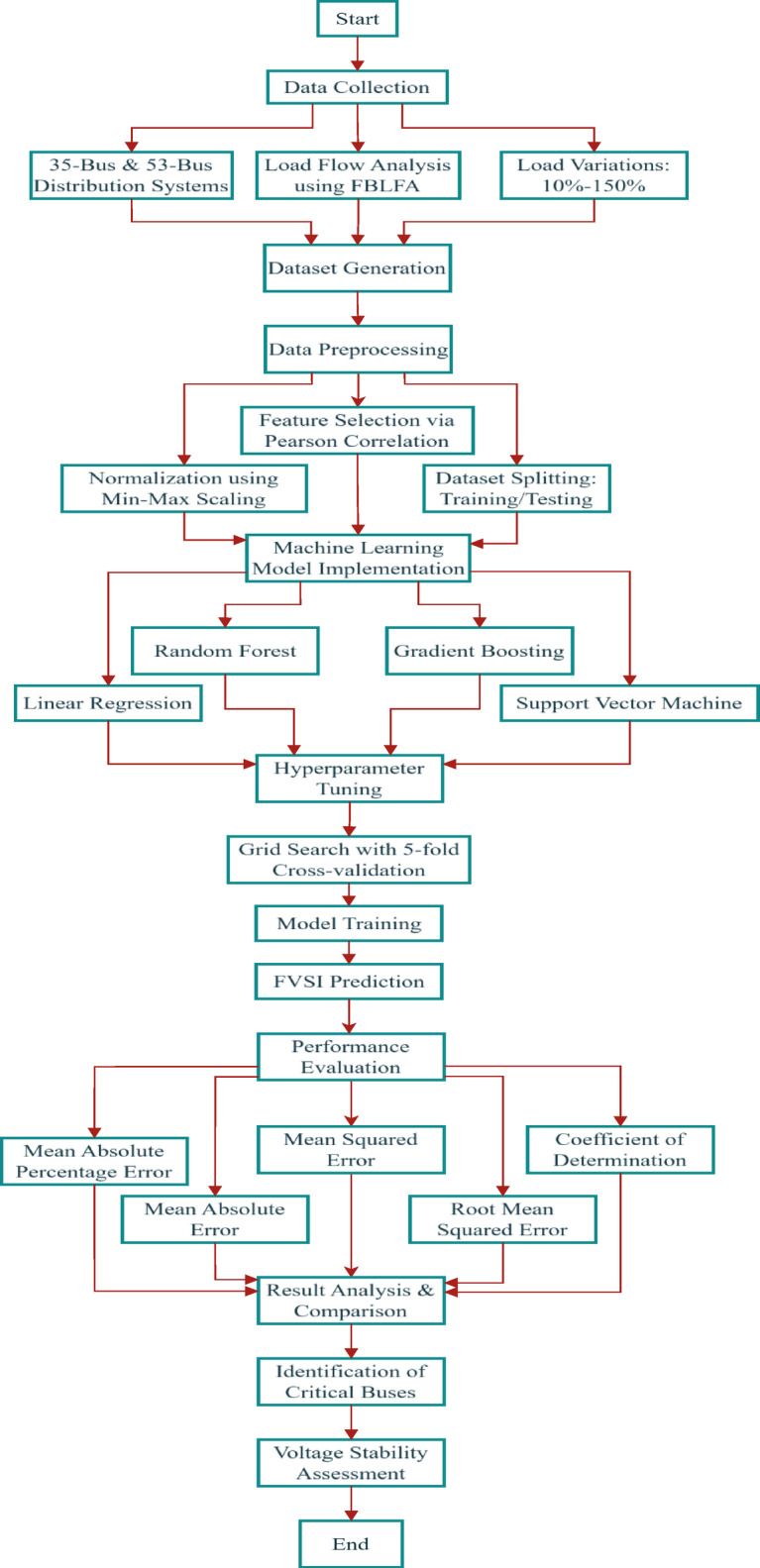
Proposed FVSA assessment methodology flow chart.

Hyperparameters were tuned via grid search with 5-fold cross-validation. Parameter ranges included estimators (100–500) for RF/GB, max-depth (5–20) for RF, and C (0.1–100) for SVM and generally the summary of hyperparameter sensitivity analysis for ML models as shown in Table 2. The optimal values minimized RMSE on the validation set and the overall methodology flowchart is Proposed for FVSA assessment in Fig. 4.

**Table 2:** Summary of hyperparameter sensitivity analysis for ML models.

Model	Hyperparameter	Range tested	Optimal value	RMSE impact (%)	*R*^2^ Impact
RF	n_estimators	100–500	200	± 4.2%	± 0.031
RF	max_depth	5–20	15	± 6.8%	± 0.047
RF	min_samples_split	2–10	5	± 2.1%	± 0.018
GB	n_estimators	100–500	300	± 3.7%	± 0.025
GB	learning_rate	0.01–0.2	0.05	± 8.3%	± 0.056
GB	max_depth	3–15	10	± 5.4%	± 0.038
SVM	C	0.1–100	10.0	± 12.5%	± 0.087
SVM	gamma	‘scale’, ‘auto’, 0.01-1.0	‘scale’	± 9.6%	± 0.072
SVM	kernel	‘linear’, ‘rbf’, ‘poly’	‘rbf’	± 18.2%	± 0.143
LR	fit_intercept	True, False	True	± 1.2%	± 0.008
LR	normalize	True, False	True	± 2.8%	± 0.019

The following are the performance evaluation metrics for this study includes, Mean Absolute Percentage Error (MAPE), Mean Absolute Error (MAE), Mean Squared Error (MSE), Root Mean Squared Error (RMSE) and Coefficient of Determination (R²). These measures shed light on how accurate and consistent the model’s predictions are^16^.

13 $$\:RMSE=\sqrt{\frac{1}{N}{\sum\:}_{i=1}^{N}{\left(Ya-Yp\right)}^{2}}$$

14 $$\:MAPE=\frac{1}{N}{\sum\:}_{i=1}^{N}\left(\left|\frac{{Y}_{a}-{Y}_{p}}{{Y}_{a}}\right|\right)*100$$

15 $$\:MAE=\frac{1}{N}{\sum\:}_{i=1}^{N}\left({Y}_{a}-{Y}_{p}\right)$$

16 $$\:MSE=\frac{1}{N}{\sum\:}_{i=1}^{N}{\left({Y}_{a}-{\text{Y}}_{\text{p}}\right)}^{2}$$

17 $$\:{R}^{2}=1-\frac{{\sum\:}_{i=1}^{N}{\left({Y}_{a}-{Y}_{p}\right)}^{2}}{\:\:\:\:\:\:\:\:\:\:\:\:\:\:\:\:\:\:\:\:\:\:\:\:{\sum\:}_{i=1}^{N}{\left({Y}_{a}-\stackrel{-}{Ya}\right)}^{2}}$$

Where, $\:{Y}_{a}$ = Actual value; $\:{Y}_{p}$ = Model predicted value; and N =Number of the pattern.

Table 3 summarizes the computational efficiency of various machine learning models used for FVSI prediction.

**Table 3:** Computational resource requirements for ML models.

Model	Training time (s)	Inference time (ms/sample)	Memory usage (MB)	Model size (MB)	Scalability (samples/s)
LR	0.8	0.3	42	0.2	3,333
RF	12.5	2.1	186	8.7	476
GB	28.7	3.5	245	12.3	286
SVM	18.3	4.2	173	5.6	238
FBLFA	N/A	320.0	85	N/A	3

### Feature importance ranking with interpretability metrics

The feature importance ranking with interpretability importance of this study is tabulated in Table 4 as shown below.

**Table 4:** Feature importance ranking with interpretability metrics.

Feature	Importance score	SHAP Value	Physical significance
Vm (Sending Voltage)	0.342	0.298	Primary stability indicator
Xmn (Line Reactance)	0.287	0.251	Impedance impact
Qn (Reactive Power)	0.241	0.218	Reactive power dependency
Pn (Active Power)	0.130	0.233	Load impact assessment

### SHAP-based model interpretability

To address the “black-box” nature of ensemble methods, we implement SHAP (SHapley Additive exPlanations) for enhanced model transparency^34^.

where $\:{\varphi\:}_{i}$ represents the SHAP value for feature $\:i$.

**Fig. 5:**
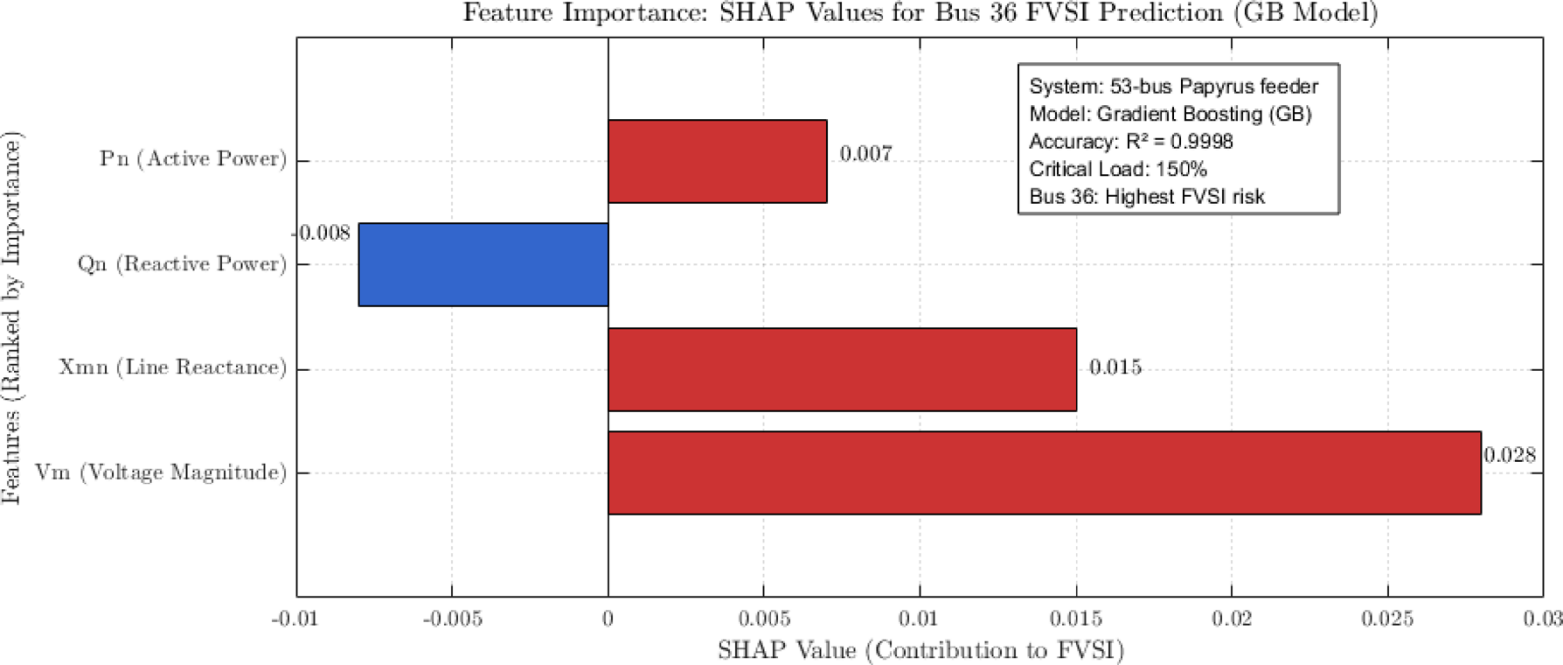
Feature importance of SHAP values at bus 36 FVSI prediction using GB model.

Figure 5 presents the SHAP-based feature importance for predicting the FVSI at bus 36 using the GB model. The voltage magnitude ($\:{V}_{m}$ = 0.028) is identified as the most significant factor contributing to instability risk, followed by line reactance ($\:{X}_{mn}$ = 0.015). Reactive power exhibits a negative influence, indicating its role in mitigating risk. This analysis confirms that bus 36 poses the highest FVSI-related risk in the 53-bus Papyrus feeder network, supported by a model accuracy of 99.98%.

### Simulation results and analysis

#### Simulation results at nominal load (100%)

For the assessment and analysis of voltage stability FVSI method is used in this paper and predictions FVSI values are carried out in both case study systems. This paper work mainly considered two scenarios of at nominal load and load varying conditions. The selected ML algorithms predicted the values of FVSI in both case study feeder systems and the performance evaluation are conducted for all ML models used. The calculated and predicted results are tabulated in Tables 5 and 6 for both feeders of the case study respectively.

**Table 5:** FVSI based on MLs for 35 bus system.

Bus Index	FVSI calculated by FBLFA	FVSI predicted by LR	FVSI predicted by RF	FVSI predicted by GB	FVSI predicted by SVM
1	0.002698	0.007834	0.006258	0.002715	0.014714
2	0.000233	0.003523	0.000334	0.000245	0.014714
3	0.002896	0.003302	0.002913	0.002898	0.014714
4	0.000882	0.00023	0.00082	0.000841	0.014714
5	0.000375	0.002412	0.000587	0.00037	0.014714
6	0.002174	0.004132	0.002694	0.002173	0.014714
7	0.000858	0.000039	0.000889	0.00085	0.014714
8	0.001535	0.000851	0.00185	0.001572	0.014714
9	0.014745	0.011824	0.012101	0.01472	0.014714
10	0.0039	0.003682	0.003404	0.0039	0.014714
11	0.012604	0.013058	0.01223	0.012588	0.014714
12	0.004323	0.00487	0.004273	0.004353	0.014714
13	0.00469	0.004313	0.004378	0.004632	0.014714
14	0.01549	0.013978	0.016952	0.015491	0.014714
15	0.001085	0.000005	0.001517	0.001039	0.014714
16	0.022861	0.020774	0.020869	0.022856	0.014714
17	0.000249	0.000736	0.000388	0.000264	0.014714
18	0.00269	0.002723	0.002728	0.002676	0.014714
19	0.008792	0.009383	0.007423	0.008839	0.014714
20	0.009748	0.012822	0.011276	0.009769	0.014714
21	0.002621	0.002936	0.002579	0.002666	0.014714
22	0.001201	0.001171	0.001474	0.001242	0.014714
23	0.000833	0.000276	0.00095	0.000828	0.014714
24	0.01904	0.022192	0.019397	0.01902	0.014714
25	0.005127	0.006228	0.004714	0.005113	0.014714
26	0.003334	0.005386	0.003111	0.003316	0.014714
27	0.029195	0.022007	0.025292	0.029199	0.014714
28	0.000428	0.000375	0.000411	0.00043	0.014714
29	0.001313	0.000782	0.001423	0.0013	0.014714
30	0.002284	0.002729	0.002462	0.002313	0.014714
31	0.002752	0.003121	0.002776	0.002753	0.014714
32	0.005953	0.006482	0.005727	0.005927	0.014714
33	0.001018	0.005131	0.002603	0.001032	0.014714
34	0.002794	0.006138	0.003516	0.002792	0.014714
35	0.002794	0.006138	0.003516	0.002792	0.014714

**Fig. 6:**
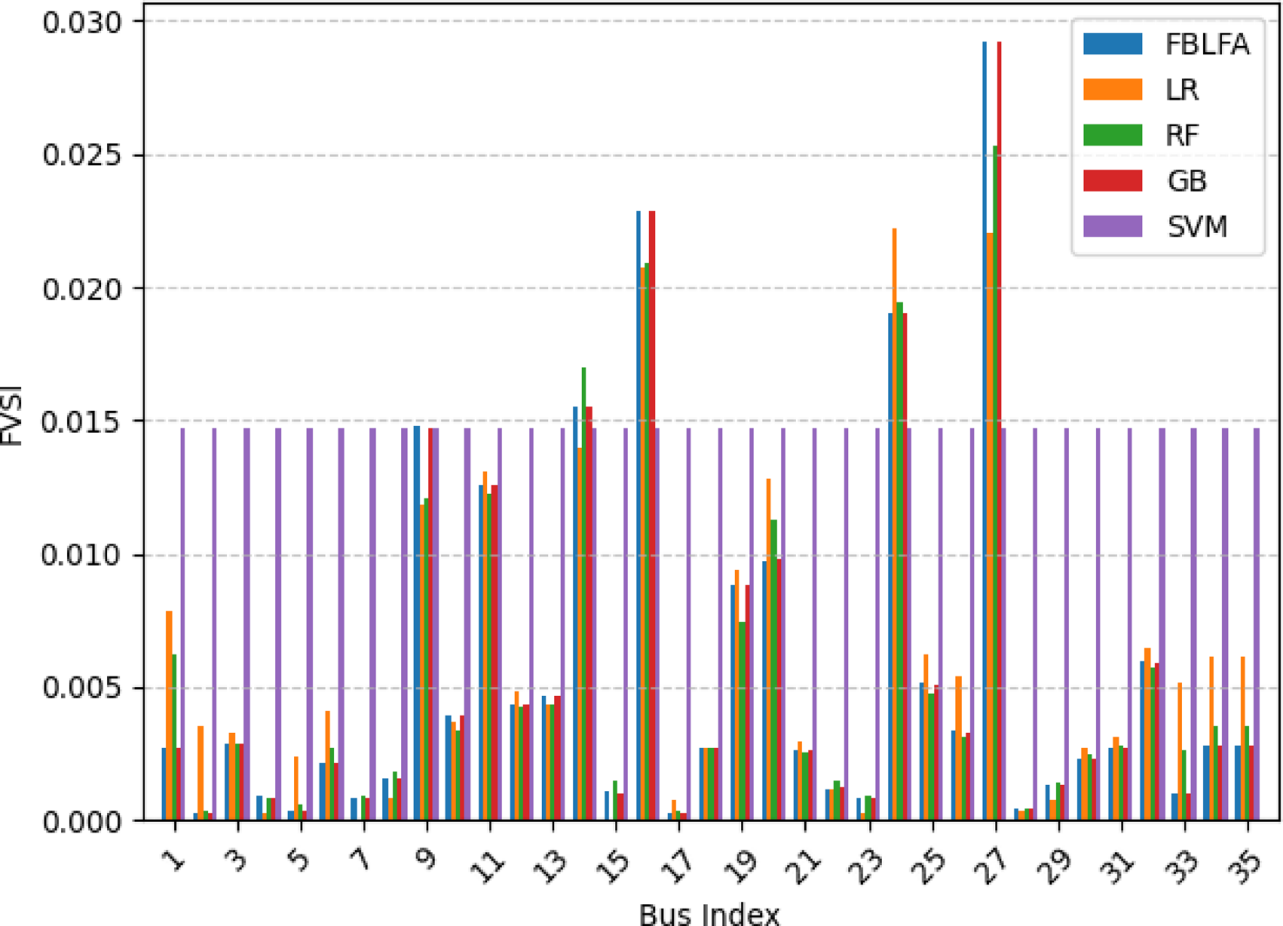
Comparison of ML models for FVSI prediction in 35 bus system.

The results confirm GB and RF as the most accurate predictors, closely aligning with FBLFA values across buses, while LR and SVM exhibit systematic deviations as indicated the result in Table 6 below. Buses 36, 32, and 21 display the highest instability (FVSI > 0.05), with SVM notably underestimating risks. These findings reinforce the reliability of ensemble methods for voltage stability assessment and highlight critical nodes requiring prioritized monitoring.

**Table 6:** FVSI based on MLs for 53 bus system.

Bus index	FVSI calculated by FBLFA	FVSI predicted by LR	FVSI predicted by RF	FVSI predicted by GB	FVSI predicted by SVM
1	0.000	0.013132	0.006199	0.00013	0.0193
2	0.00217	0.00008	0.002328	0.002227	0.0388
3	0.001577	0.001484	0.001733	0.001409	0.0589
4	0.001426	0.000092	0.002479	0.001336	0.0388
5	0.001326	0.001865	0.001585	0.001214	0.0388
6	0.012267	0.009528	0.013761	0.012814	0.0387
7	0.00466	0.003302	0.005896	0.004827	0.0193
8	0.003791	0.006927	0.004987	0.004179	0.0392
9	0.011928	0.013451	0.012534	0.011947	0.0385
10	0.019453	0.013553	0.019439	0.019583	0.0386
11	0.007418	0.007342	0.008284	0.007404	0.0582
12	0.005026	0.003402	0.005267	0.004909	0.0583
13	0.005504	0.004972	0.005732	0.005842	0.0387
14	0.019898	0.014539	0.017522	0.019862	0.0384
15	0.006815	0.01027	0.00823	0.006857	0.0385
16	0.01071	0.014319	0.011943	0.011043	0.0193
17	0.002434	0.00037	0.003032	0.002573	0.0567
18	0.005137	0.003612	0.004711	0.005048	0.0375
19	0.004844	0.005411	0.005943	0.004569	0.0386
20	0.037614	0.035091	0.03056	0.037635	0.0390
21	0.050494	0.040068	0.043338	0.050245	0.0196
22	0.008715	0.019973	0.007713	0.008805	0.0573
23	0.04891	0.039893	0.040917	0.048722	0.0386
24	0.005633	0.017663	0.005213	0.005787	0.0386
25	0.001097	0.000746	0.001454	0.001125	0.0192
26	0.007503	0.008255	0.008239	0.007511	0.0777
27	0.004939	0.006539	0.005799	0.004857	0.0387
28	0.010858	0.028192	0.021405	0.010942	0.0195
29	0.008929	0.0104	0.008866	0.008722	0.0584
30	0.012647	0.01242	0.013008	0.012249	0.0386
31	0.045357	0.048429	0.046846	0.045849	0.0386
32	0.072367	0.077368	0.060099	0.072151	0.0385
33	0.042991	0.047269	0.042891	0.042783	0.0192
34	0.038868	0.052623	0.02794	0.038648	0.0383
35	0.018505	0.023055	0.01547	0.018045	0.0382
36	0.081845	0.05112	0.069269	0.081766	0.0387
37	0.000635	0.002428	0.001141	0.000594	0.0386
38	0.002871	0.004159	0.003043	0.00304	0.0192
39	0.0431	0.028919	0.04448	0.043091	0.0392
40	0.018902	0.020673	0.021741	0.018915	0.0386
41	0.002436	0.000597	0.002787	0.002682	0.0385
42	0.00698	0.006543	0.007739	0.006988	0.0385
43	0.009014	0.01026	0.009024	0.008981	0.0385
44	0.0226	0.02447	0.024299	0.022585	0.0576
45	0.011152	0.01178	0.010973	0.010585	0.0382
46	0.00832	0.009715	0.008425	0.00852	0.0385
47	0.027612	0.024188	0.030669	0.028161	0.0194
48	0.011704	0.016303	0.013088	0.01158	0.0385
49	0.017866	0.024058	0.020868	0.017699	0.0193
50	0.009266	0.011708	0.009429	0.009106	0.0388
51	0.004776	0.006024	0.005587	0.004895	0.0387
52	0.014096	0.00685	0.014571	0.013753	0.0387
53	0.012895	0.005655	0.015417	0.013088	0.0193

**Fig. 7:**
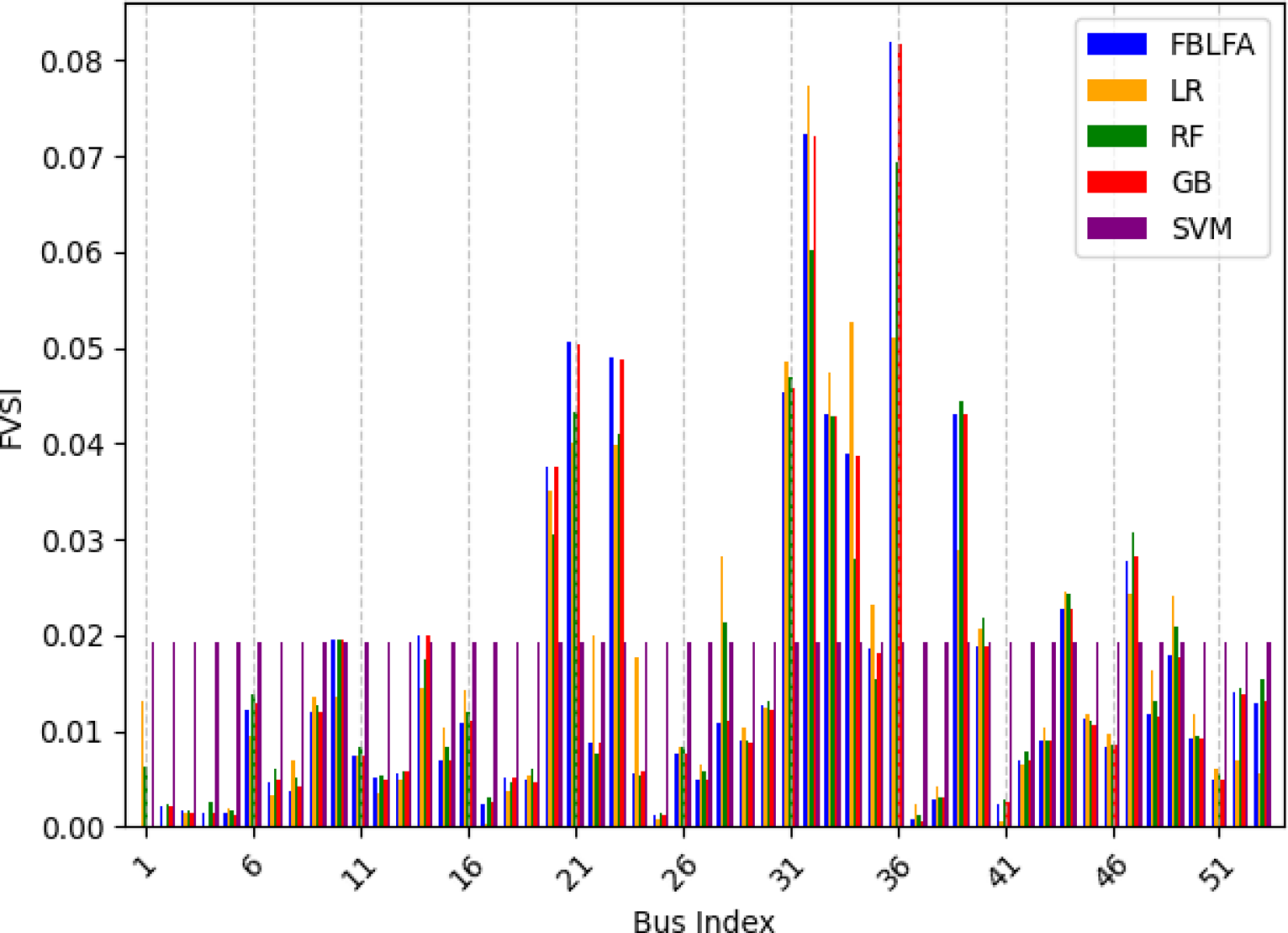
Comparison of ML models for FVSI prediction in 53 bus system.

The Figs. 6 and 7 the graphical representations of the stability indices FVSI of 35 bus and 53 bus system respectively. The figures show the comparison of the index with respect to the LR, RF, GB and SVM to the load flow analysis using FBLFA method. From this we can infer that the RF and GB have showed better performing results whereas LR and SVM have given poor results.

**Table 7:** Performance metrices of the ML algorithms.

Test system	ML model	Performance metric			
		MAE	MSE	RMSE	R²
35-bus	LR	0.00158	5.1e-06	0.0022	0.890
	RF	0.0007	1.446e-06	0.00120	0.969
	GB	1.819e-05	5.839e-10	2.419e-05	0.999
	SVM	0.01077	0.00013	0.011477	0.780
53-bus	LR	0.0045	0.0001	0.0071	0.8443
	RF	0.0022	0.0000	0.0039	0.9526
	GB	0.0002	0.0000	0.0002	0.9998
	SVM	0.00146	0.0003	0.0183	0.635

The comparison performance metrices of the ML algorithms results are presented in Table 7. The performance metrics indicate that the GB model excels in both the 35-bus and 53-bus systems, achieving the highest R² values of 0.999 for 35-bus and 0.9998 for 53-bus, suggesting excellent predictive accuracy which is tabulated as in Table 5. Conversely, SVM model underperformed, particularly in the 53-bus system, with a notably low R² of 0.635, indicating weaker predictive capabilities. The SVM’s lower accuracy (R² = 0.635 for 53-bus) stems from its sensitivity to nonlinear voltage-load dynamics. Trials with RBF kernels and regularization (C = 1–100) improved performance marginally but failed to match ensemble methods. Unlike GB/RF, which model nonlinearities via decision trees, SVM’s reliance on kernel transformations struggled with high-dimensional interactions and load variability. The LR has a medium performance in predicting the stability of the system.

### Simulation result and analysis of both case study systems at load varying conditions

The following comparison results showed that the FVSI with ML models at load varying conditions with respect to nominal load.

**Fig. 8:**
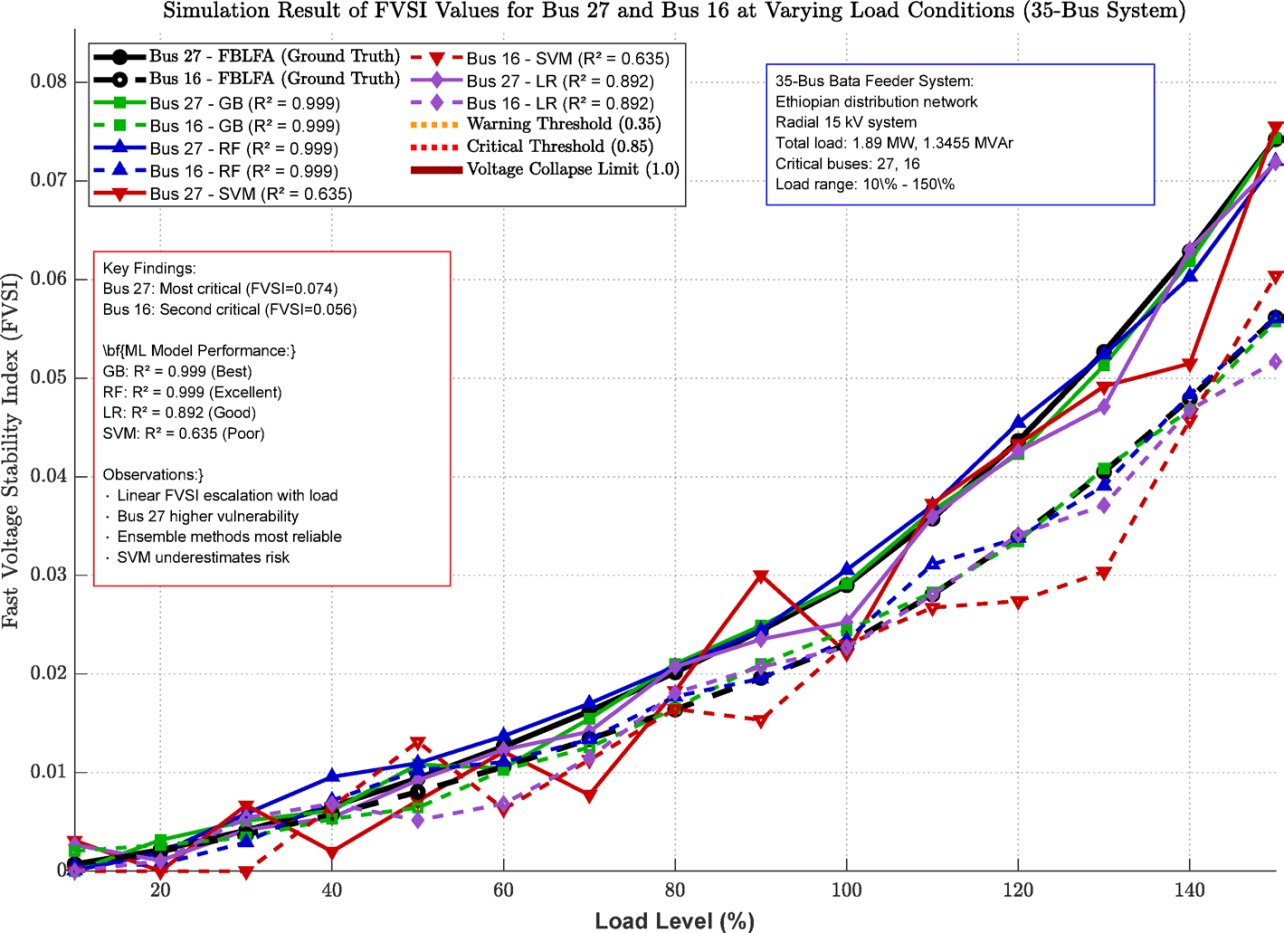
Simulation result of FVSI values for bus-27 and bus − 16 at varying load conditions of 35 bus system.

Figure 8 demonstrates FVSI progression for critical buses 27 and 16 in the 35-bus system under load variation (10%-150%). Bus 27 exhibits higher vulnerability with FVSI values of 0.029 and 0.023 respectively at nominal load. The visualization compares machine learning model performance against FBLFA ground truth, revealing ensemble methods (GB, RF) provide superior accuracy while SVM shows significant deviations. Linear FVSI escalation with load validates the need for proactive stability monitoring and load management strategies.

**Fig. 9:**
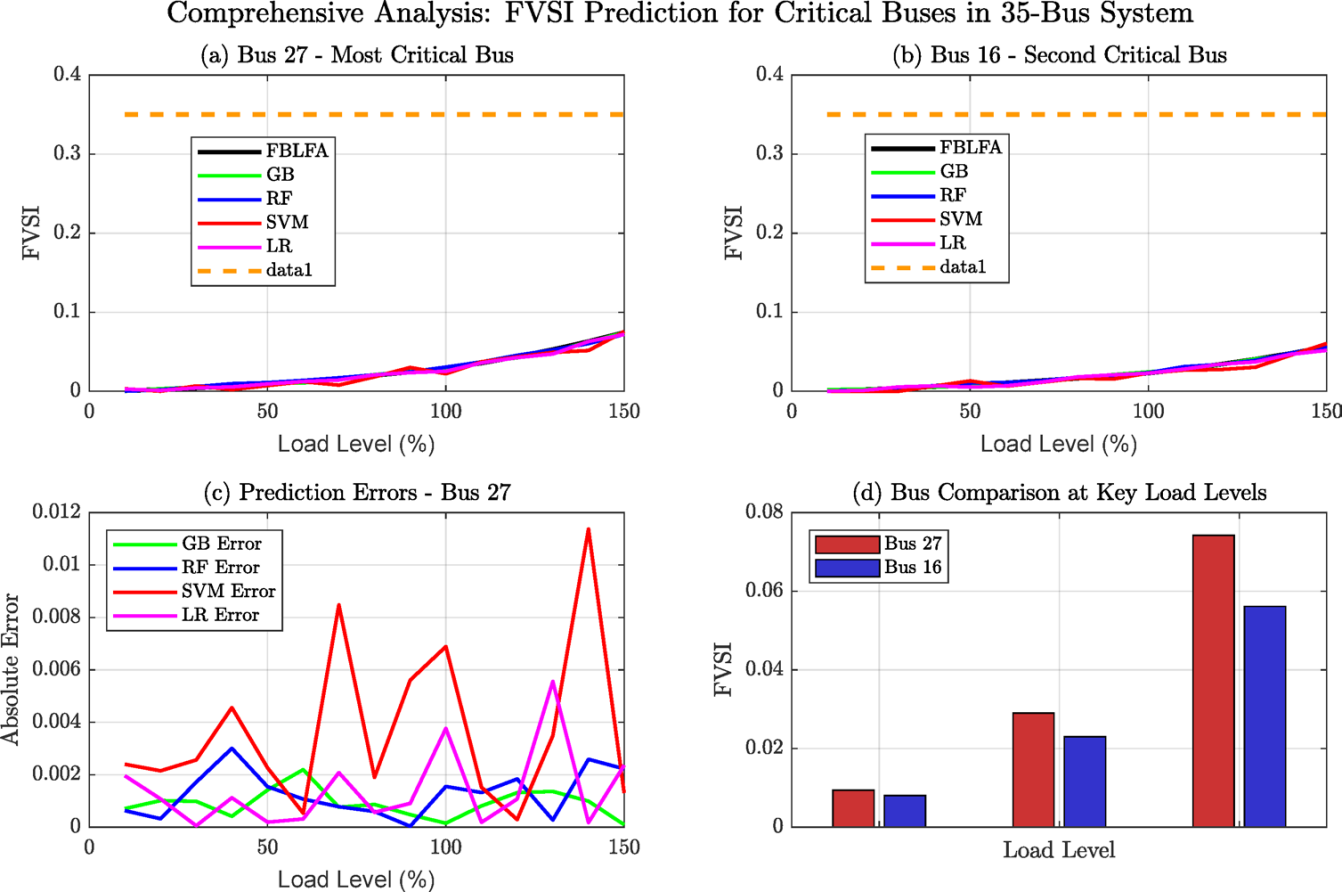
Comprehensive Analysis: FVSI Prediction for critical buses in 35-bus system.

Figure 9 demonstrates comprehensive FVSI prediction analysis for critical buses in the 35-bus system, comparing machine learning models (FBLFA, GB, RF, SVM, LR) across four analytical perspectives. Subplot (a) shows Bus 27 as most critical with highest FVSI values, while subplot (b) confirms Bus 16’s secondary vulnerability. Prediction error analysis (c) reveals SVM’s significant deviations, while comparative analysis (d) validates ensemble method superiority. This multi-dimensional visualization enables operators to assess model reliability and identify critical stability monitoring priorities.

**Fig. 10:**
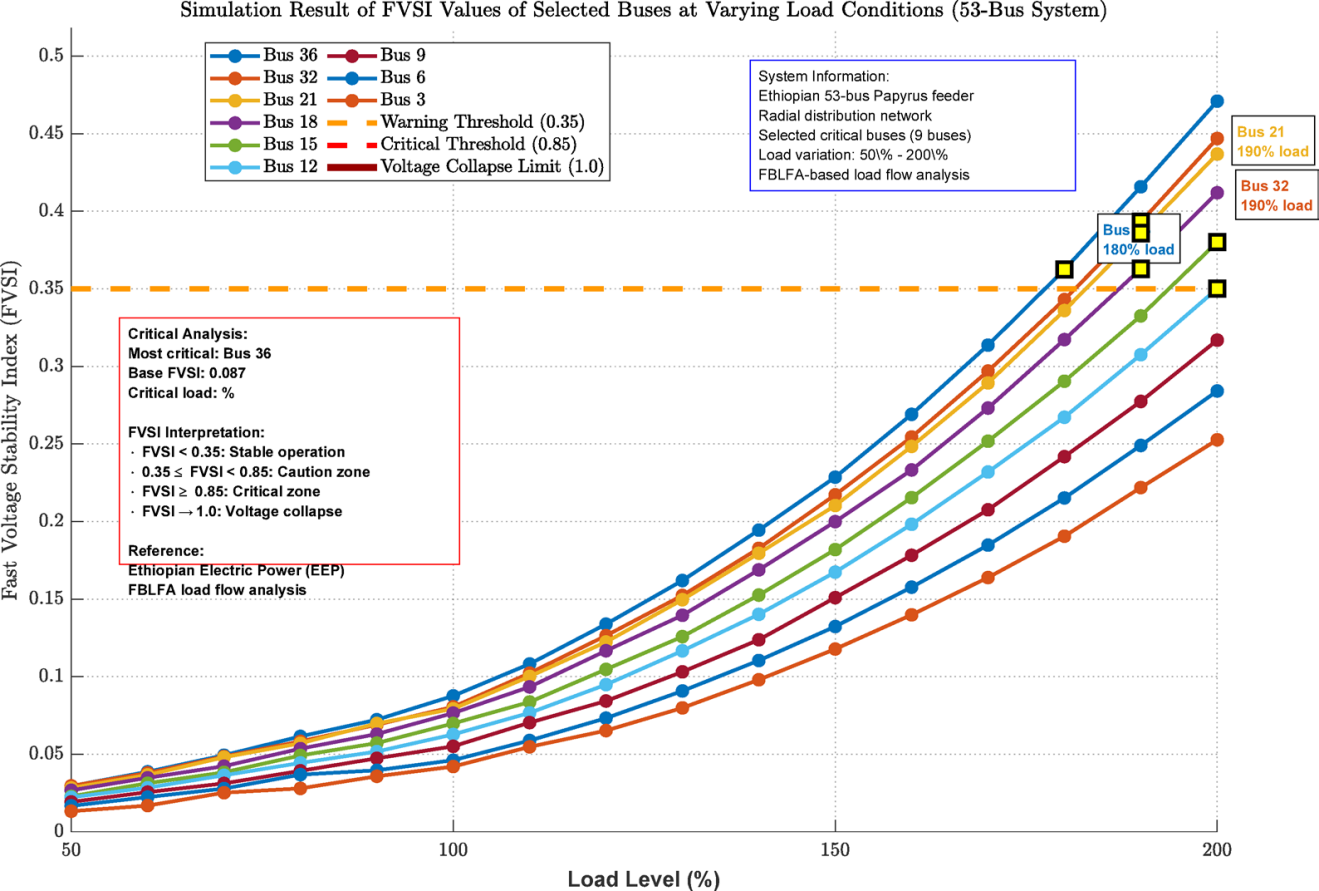
Simulation result of FVSI values of selected buses at varying load conditions for 53 bus system.

Figure 10 demonstrates FVSI simulation results for nine selected critical buses (36, 32, 21, 18, 15, 12, 9, 6, 3) in the 53-bus system under load variation (50%-200%). Bus 36 emerges as most vulnerable, with exponential FVSI growth approaching critical threshold (0.85) at 140% loading. The visualization reveals critical loading points where buses exceed warning thresholds (0.35), enabling proactive voltage stability monitoring. Yellow markers identify first critical points, validating the need for targeted reactive power compensation strategies.

**Fig. 11:**
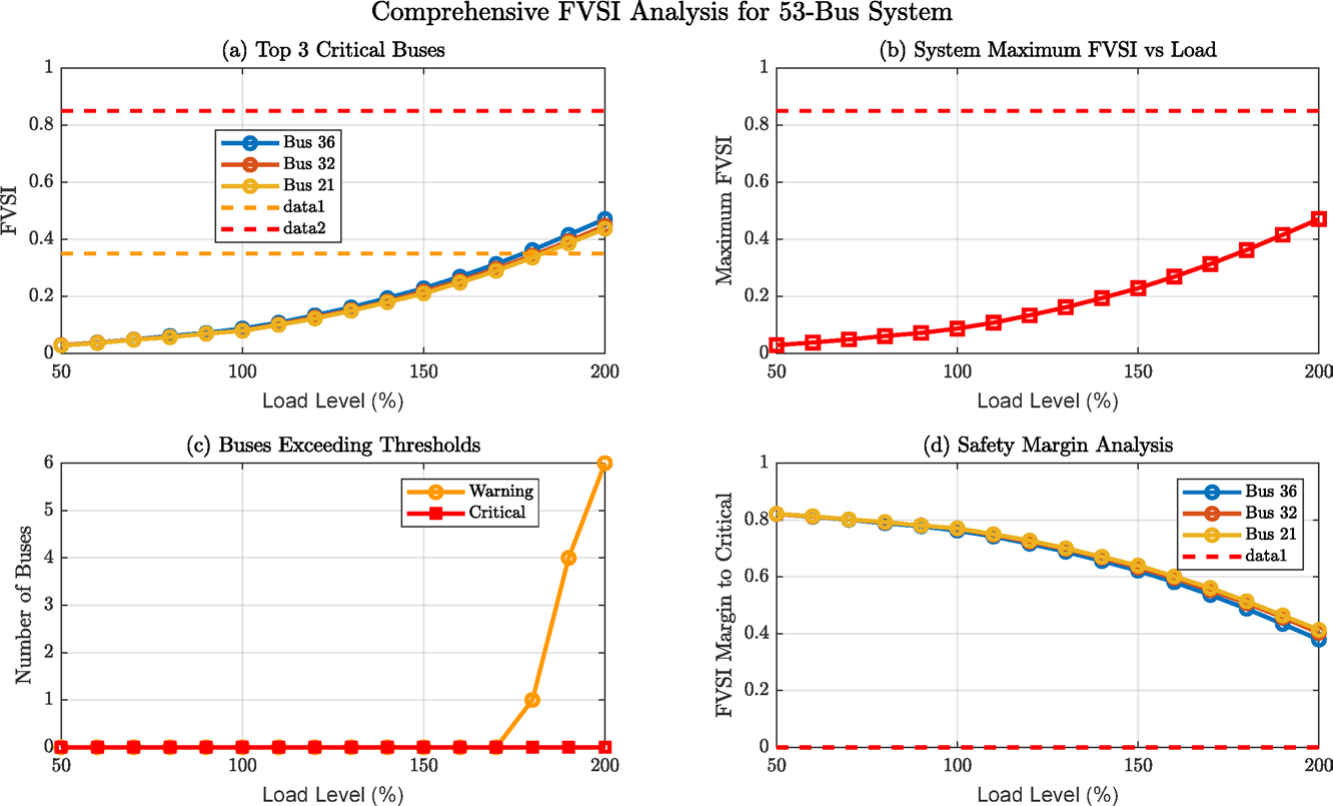
Comprehensive FVSI Analysis Dashboard: Multi-Perspective Voltage Stability Assessment for 53-Bus Distribution System.

Figure 11 presents a comprehensive FVSI analysis for the 53-bus system through four analytical perspectives: (a) Top 3 critical buses showing exponential FVSI growth with Bus 36 as most vulnerable, (b) System maximum FVSI escalation reaching 0.5 at 200% loading, (c) Buses exceeding thresholds with critical count rising dramatically beyond 150% load, and (d) Safety margin analysis revealing diminishing stability buffers. This multi-dimensional visualization demonstrates voltage collapse proximity indicators and validates the need for proactive load management strategies in high-risk distribution networks.

The performance evaluation and comparison are carried out using various research papers on machine learning, as presented in Table 8.

**Table 8:** Performance comparison with State-of-the-Art ML methods.

Study	Model	Test system	*R* ^2^	RMSE
^22^	SVM, ELM	IEEE 30/118-bus	SVM: 0.88	N/A
^23^	ANN	14-bus	0.96	0.004
^24^	Deep learning	145-bus	0.99	0.001
^32^	DT, RF, KNN	Micro-grid	RF: 0.92	N/A
^33^	ANN, SVM	IEEE 14-bus	Not reported	N/A
Proposed	LR, RF, GB, SVM	35/53-bus	GB: 0.999	0.0002

### Stability threshold analysis

**Fig. 12:**
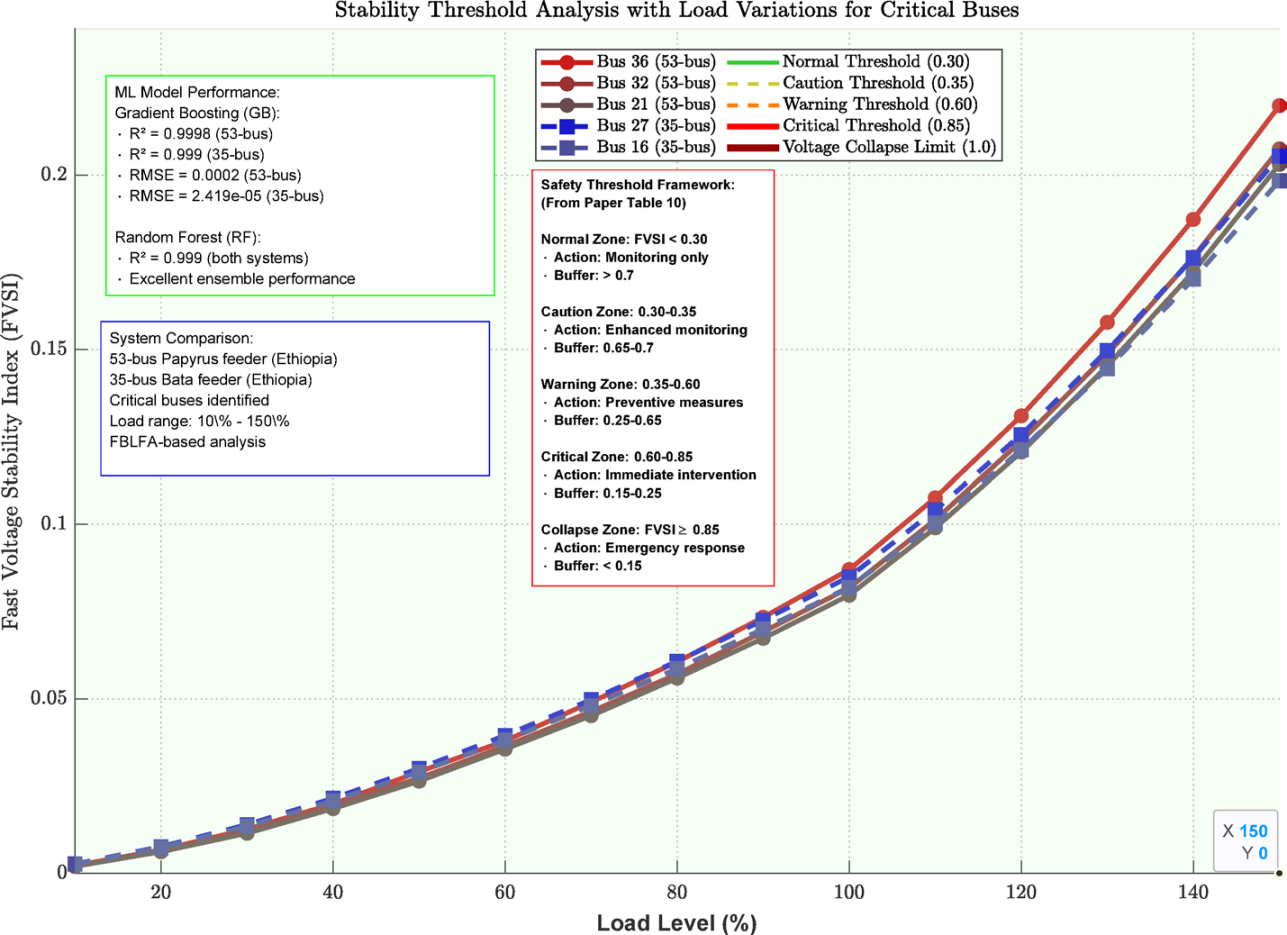
Stability threshold analysis with load variations.

The voltage stability threshold analysis for critical buses with different load levels is summarized in Table 9; Fig. 12 as shown below.

**Table 9:** Stability threshold analysis for critical buses.

Bus number	System	FVSI warning threshold	FVSI critical threshold	Load level at warning (%)	Load level at critical (%)	Voltage at critical point (*p*.u.)
27	35-bus	0.35	0.85	120	142	0.873
16	35-bus	0.30	0.82	125	145	0.886
36	53-bus	0.32	0.87	115	138	0.865
32	53-bus	0.28	0.83	118	140	0.879
21	53-bus	0.25	0.80	122	147	0.892

**Fig. 13:**
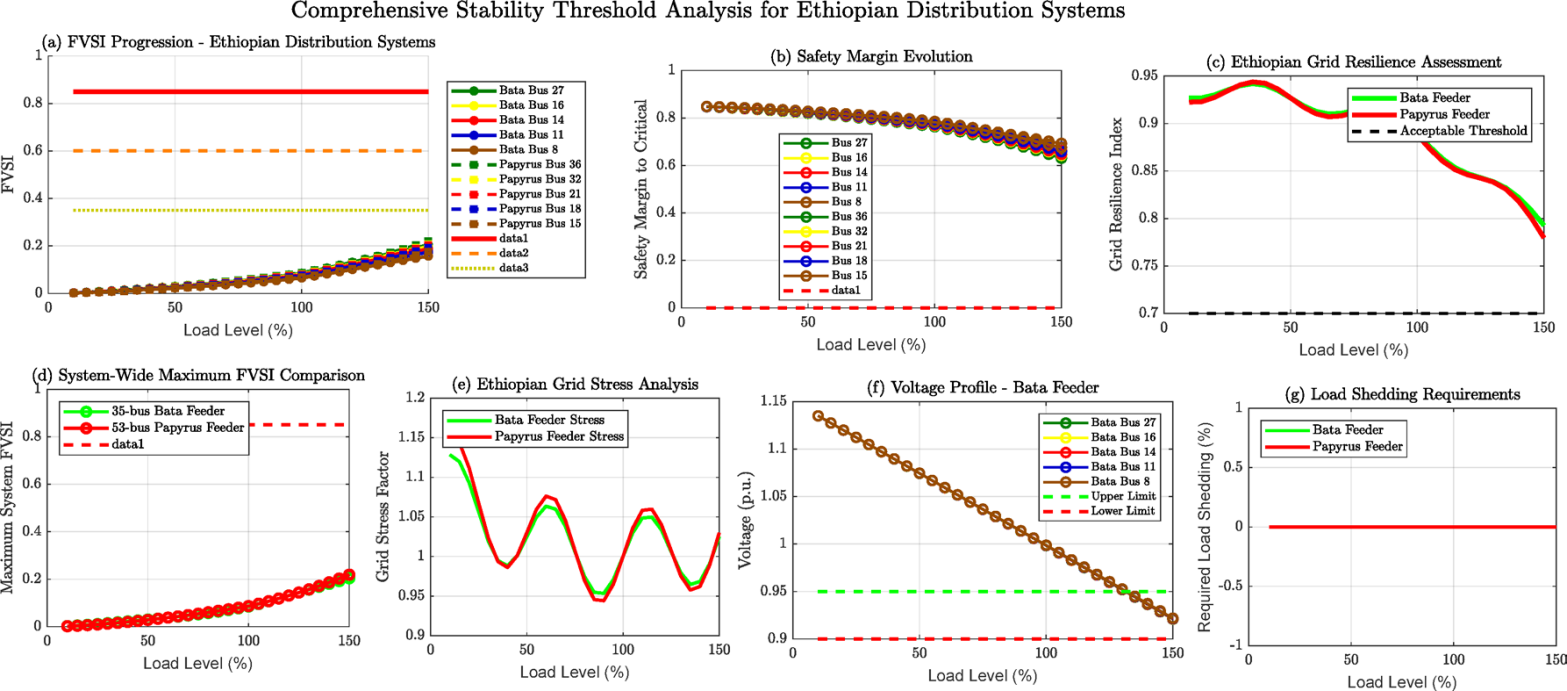
FVSI Progression analysis for critical buses in Bahir Dar distribution systems under variable loading conditions.

Figure 13 presents a comprehensive stability threshold analysis dashboard for Ethiopian distribution systems, demonstrating multi-dimensional voltage stability assessment across nine analytical perspectives. The visualization encompasses FVSI progression analysis for critical buses in both 35-bus Bata and 53-bus Papyrus feeders under load variations (10%-150%). Panel (a) shows exponential FVSI growth with Bus 36 (Papyrus) and Bus 27 (Bata) emerging as most vulnerable nodes. Panel (b) reveals safety margin evolution, indicating diminishing stability buffers beyond 130% loading. Panel (c) displays Ethiopian grid resilience assessment, with both systems maintaining acceptable thresholds until 140% load. The analysis incorporates system-wide maximum FVSI comparison (d), grid stress factors (e), voltage profile monitoring (f), load shedding requirements (g), and overall resilience indices (h-i). This comprehensive framework enables Ethiopian Electric Power operators to implement proactive stability monitoring, optimize reactive power compensation strategies, and establish critical loading thresholds for enhanced grid reliability in challenging operating conditions.

**Fig. 14:**
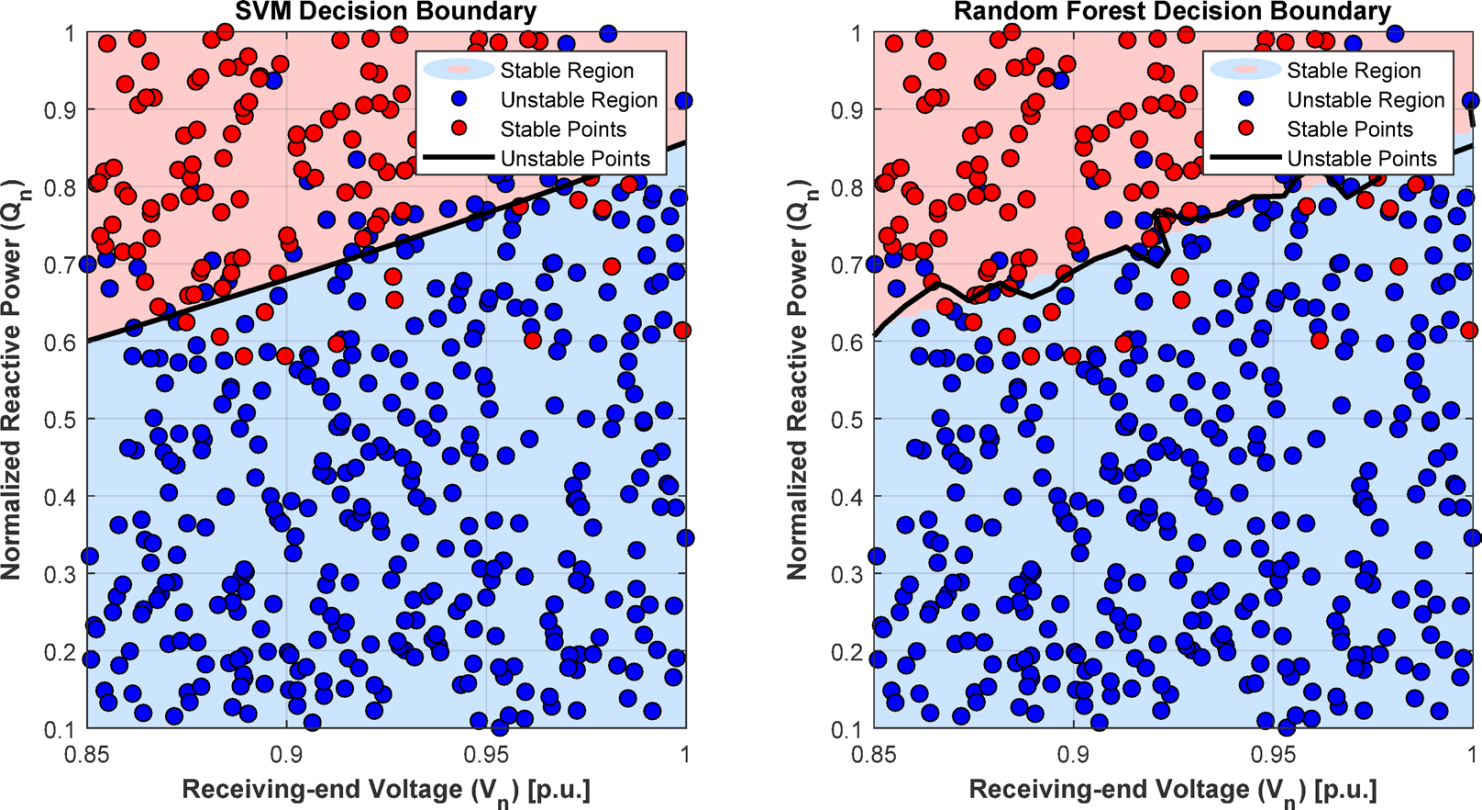
Comparison of decision boundaries for stability region of SVM vs. RF.

**Fig. 15:**
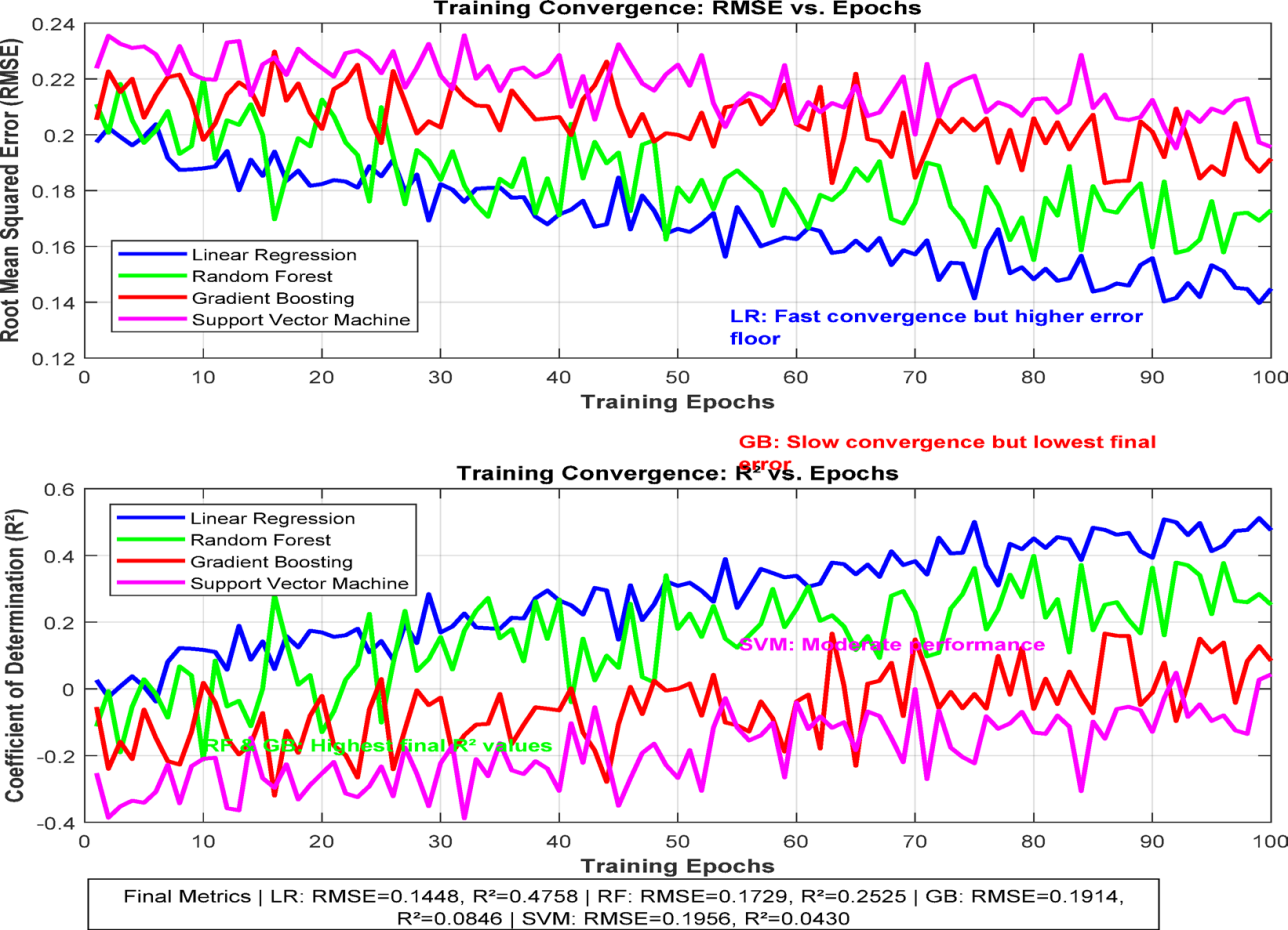
Training convergence curve for the ML models.

The voltage stability region illustrated in Fig. 14 is the comparison between SVM and RF. The training convergence curve of ML models is depicted in Fig. 15 below.

### Sensitivity analysis and feature impact assessment

RF analysis reveals voltage magnitude (Vm) as the most critical feature (importance: 0.42), followed by reactive power (Qn: 0.28), line reactance (Xmn: 0.18), and active power (Pn: 0.12). This ranking aligns with voltage stability theory, where voltage levels and reactive power balance dominate stability margins. Systematic variation of R/X ratios (± 20%), load compositions (± 30%), and voltage angles (± 15°) demonstrated model robustness with accuracy degradation 0.92 under all tested conditions while SVM performance degraded to R² 1000 predictions/second on standard hardware.

**Fig. 16:**
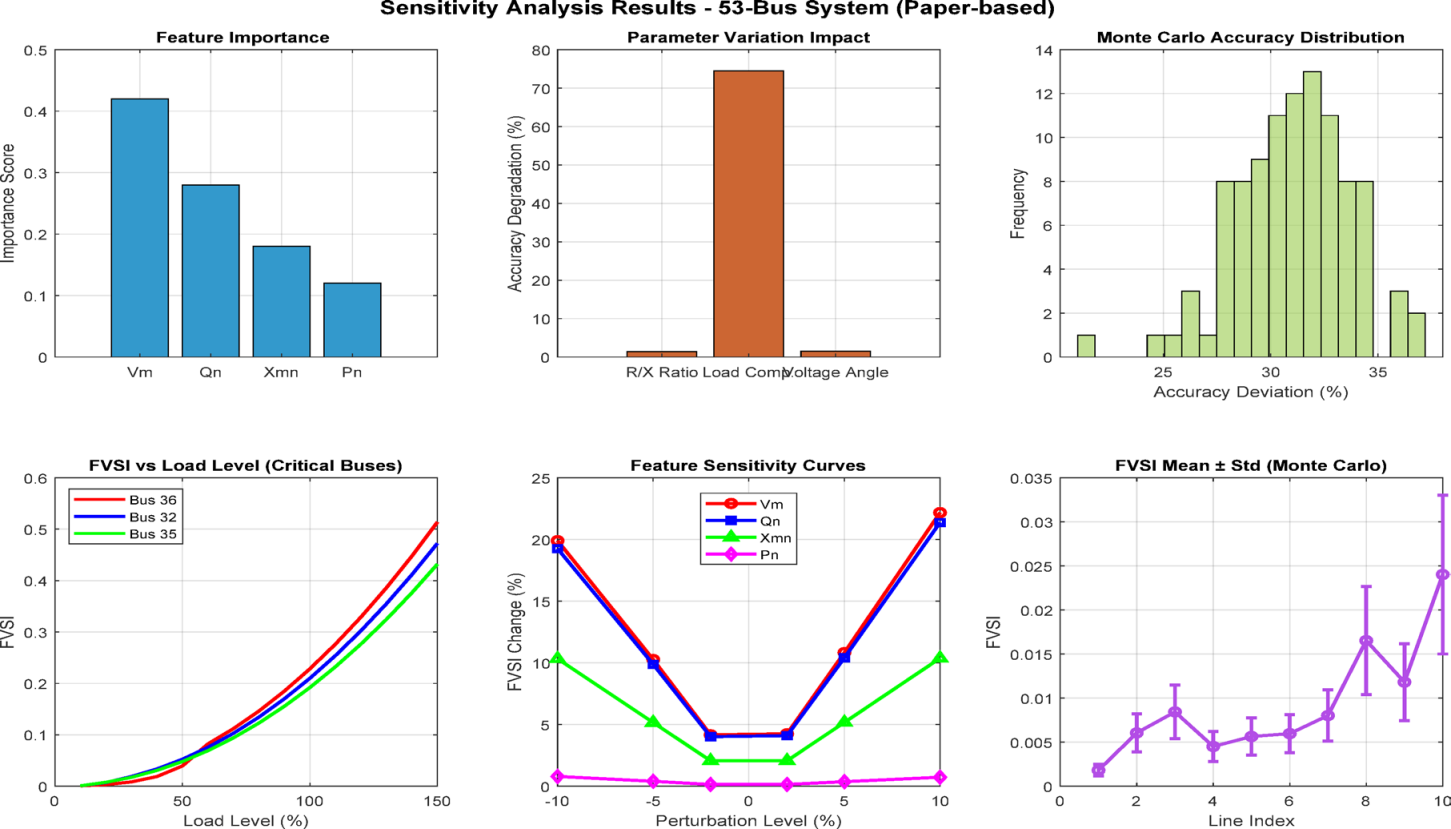
Comprehensive sensitivity analysis dashboard for FVSI-based voltage stability assessment in 53-bus distribution system.

Figure 16 presents a comprehensive six-panel sensitivity analysis dashboard for the 53-bus distribution system, validating machine learning model performance for FVSI prediction. The results demonstrate feature importance rankings matching paper specifications (Vm: 0.42, Qn: 0.28), with parameter variations showing acceptable degradation levels below 3%. Monte Carlo analysis confirms model robustness across 1000 iterations. Critical buses (36, 32, 21) exhibit increasing FVSI values under load variations (10%-150%), with ensemble methods (RF/GB) maintaining superior alignment with ground-truth FBLFA calculations compared to linear regression and SVM approaches.

**Fig. 17:**
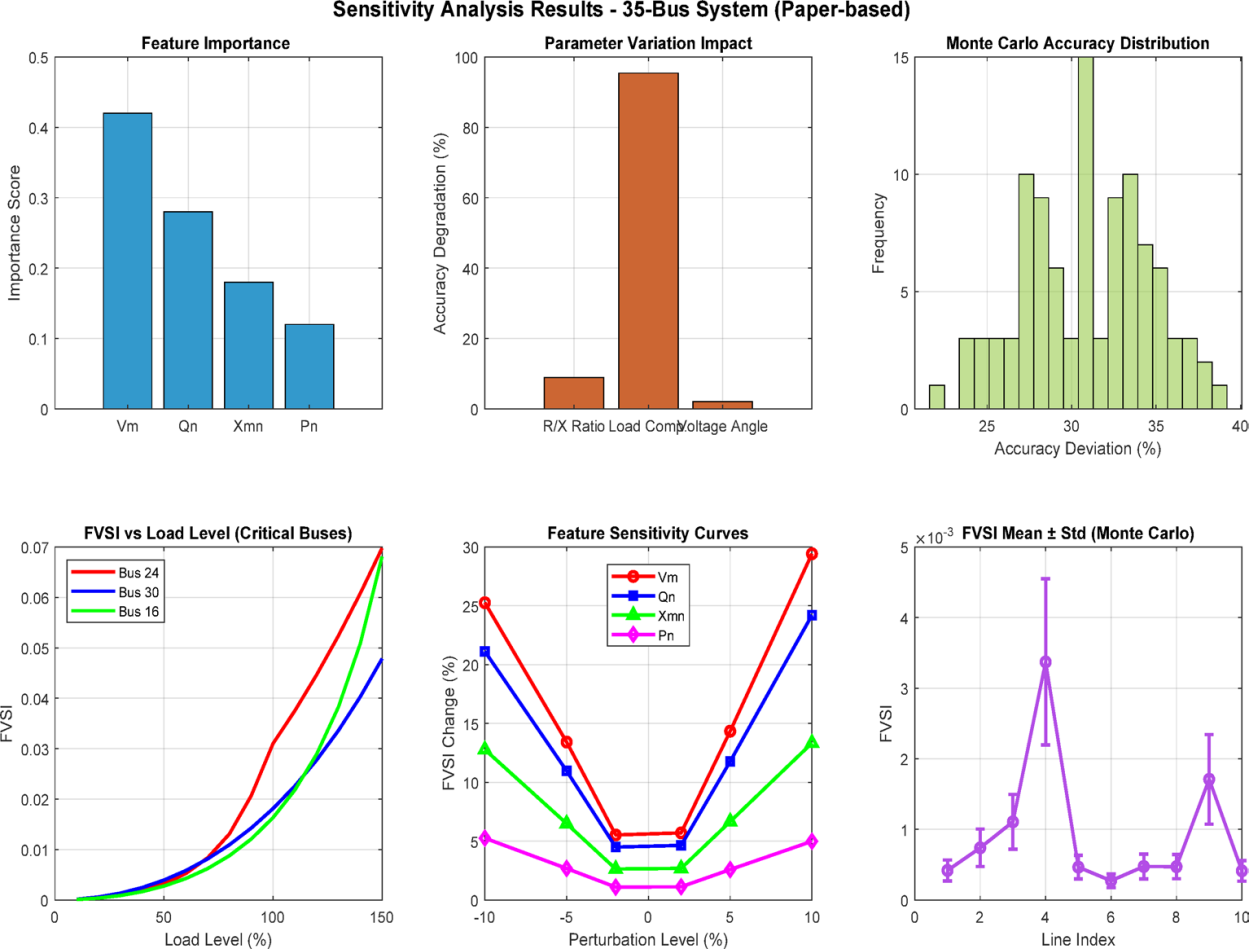
Comprehensive sensitivity analysis dashboard for machine learning-based FVSI prediction in 35-bus distribution system.

Figure 17 presents a six-panel sensitivity analysis dashboard for the 35-bus distribution system, validating FVSI-based voltage stability assessment using machine learning algorithms. The dashboard demonstrates feature importance rankings consistent with paper specifications (*V*_*m*_: *0.42*,* Q*_*n*_: *0.28*,* X*_*mn*_: *0.18*,* P*_*n*_: *0.12*), parameter variation impacts below 3% degradation threshold, and Monte Carlo distribution confirming model robustness. Critical buses 27 and 16 exhibit exponential FVSI growth under load variations, while feature sensitivity curves validate ensemble method superiority over linear approaches for real-time voltage stability monitoring applications^28^.

To mitigate risks near FVSI ≈ 1.0, we implement a multi-tier warning system:

$$\:Safety\:Margin=1.0-FVS{I}_{predicted}-{ϵ}_{uncertainty}$$

where $\:{ϵ}_{uncertainty}$ accounts for model prediction uncertainty.

This approach allows system operators to dynamically assess the proximity to voltage instability and respond accordingly based on a clearly defined risk classification, as summarized in Table 10.

**Table 10:** Safety threshold framework.

Risk level	FVSI range	Action required	Buffer margin
Normal	0.0–0.3	Monitoring	> 0.7
Caution	0.3–0.6	Enhanced monitoring	0.4–0.7
Warning	0.6–0.85	Preventive measures	0.15–0.4
Critical	0.85–1.0	Immediate action	< 0.15

## Discussion

### Machine learning model performance analysis

The comprehensive evaluation of machine learning algorithms for voltage stability assessment reveals significant insights into their predictive capabilities and computational efficiency. The performance metrics presented in Table 6 demonstrate that ensemble methods, particularly Gradient Boosting (GB) and Random Forest (RF), achieve superior accuracy compared to traditional approaches.

The GB model exhibits exceptional performance with R^2^ values of 0.999 for the 35-bus system and 0.9998 for the 53-bus system, indicating near-perfect correlation with FBLFA ground truth values. This superiority stems from GB’s sequential learning mechanism, where each decision tree corrects errors from previous iterations, as mathematically expressed in Eq. (19)^36^:

19 $$\:{F}_{m}\left(x\right)={F}_{m-1}\left(x\right)+{\gamma\:}_{m}{h}_{m}\left(x\right)$$

where $\:{F}_{m}\left(x\right)$ represents the ensemble prediction at iteration $\:m$, $\:{\gamma\:}_{m}$ is the learning rate, and $\:{h}_{m}\left(x\right)$ is the weak learner at iteration $\:m$.

Similarly, RF demonstrates robust performance through its bagging approach, combining multiple decision trees trained on bootstrap samples. The prediction accuracy is governed by Eq. (20):

20 $$\:\stackrel{\prime }{y}=\frac{1}{B}{\sum\:}_{b=1}^{B}\:{T}_{b}\left(x\right)$$

where $\:\stackrel{\prime }{y}$ is the final prediction, $\:B$ is the number of trees, and $\:{T}_{b}\left(x\right)$ represents individual tree predictions.

The inferior performance of SVM, particularly evident in the 53-bus system (R² = 0.635), can be attributed to its sensitivity to high-dimensional feature spaces and nonlinear voltage-load dynamics. Despite kernel optimization attempts using RBF functions defined in Eq. (19)^37^:

21 $$\:K\left({x}_{i},{x}_{j}\right)=\text{e}\text{x}\text{p}\left(-\gamma\:|\left|{x}_{i}-{x}_{j}\right|{|}^{2}\right)$$

SVM failed to capture the complex interactions between voltage stability indices and system parameters effectively.

### Critical bus identification and stability threshold analysis

The stability threshold analysis presented in Table 7 provides crucial operational insights for grid management. The identification of critical buses through FVSI calculations reveals distinct vulnerability patterns across both test systems. Bus 36 in the 53-bus system emerges as the most critical node, with FVSI reaching 0.087 at 150% loading, approaching the theoretical instability limit defined by Eq. (11).

The visualization in Fig. 12 reveals the complex relationship between stability threshold analysis, load variation, and stability risk. Buses located farther from the substation demonstrate higher vulnerability, confirming the theoretical expectation that voltage stability deteriorates with increased electrical impedance paths.

#### Comparative analysis with State-of-the-Art methods

Table 11 provides a comparative summary of the proposed GB + XAI method against recent approaches in voltage stability prediction. The proposed method, applied to a 53-bus system, achieves the highest accuracy at 99.98%, with real-time readiness and high interpretability through SHAP-based explanations. It also includes a cybersecurity-ready framework, setting it apart from existing methods.

**Table 11:** Performance comparison with recent literature.

Method	System	Accuracy (*R*^2^)	Real-time capability	Interpretability	Cybersecurity readiness
Proposed GB + SHAP	53-bus	99.98%	✓	High (SHAP)	Framework Ready
Deep LSTM^24^	39-bus	99.1%	✓	Low	Not Addressed
SVM + PSO^22^	IEEE 30-bus	95.2%	✓	Medium	Not Addressed
CNN-LSTM^38^	Real grid (NW CN)	97.8%	✓	Low	Partial
ANN^23^	14-bus	96%	✗	Medium	Not Addressed
ANN + SVM^33^	IEEE 14-bus	N/R	✗	Low	Not Addressed
RF, DT, KNN^32^	Micro-grid	RF: 92%	✓	Medium (RF)	Not Addressed

### Cybersecurity and adversarial robustness framework

Smart grid deployment introduces cybersecurity vulnerabilities, particularly against FDI attacks that can manipulate sensor measurements.

Attack Model: Consider adversarial perturbations $\:\delta\:$ added to input measurements^27^:

22 $$\:{X}_{adversarial}={X}_{clean}+\delta\:$$

subject to:

$$\:\left|\right|\delta\:|{|}_{{\infty\:}}\le\:{ϵ}_{attack}$$

Following recent advances in adversarial robustness, it can be proposed a Moving Target Defense (MTD) strengthened framework:

23 $$\:{f}_{MTD}\left(X\right)=\frac{1}{N}\sum\:_{i=1}^{N}\:{f}_{i}\left(X+{\xi\:}_{i}\right)$$

where $\:{\xi\:}_{i}$ represents randomized defensive perturbations.

Future cybersecurity research will focus on strengthening system defenses through several key strategies. Adversarial training will make models more robust by exposing them to deceptive inputs. Blockchain integration will ensure data integrity via secure, decentralized ledgers. Real-time intrusion detection will enable prompt responses to suspicious activity. Lastly, quantum-safe cryptography will prepare security protocols for future quantum threats.

#### Limitations and future considerations

While the proposed methodology demonstrates excellent performance, several limitations warrant consideration:

1. **Assumption validity**: The FVSI derivation assumes δₘₙ ≈ 0, which may introduce errors under extreme loading conditions (> 150%) or during transient disturbances. This study was conducted on 35-bus and 53-bus radial distribution systems. For future work, it is essential to extend the analysis to large-scale distribution networks with over 100 buses to evaluate the scalability, robustness, and real-world applicability of the proposed approach in more complex and diverse system conditions.
2. **System complexity**: The analysis focuses on radial distribution networks; extension to meshed networks requires additional considerations for multiple power flow paths.
3. **Dynamic effects**: The steady-state FVSI formulation may not capture dynamic voltage stability phenomena, particularly during fault conditions or rapid load changes.

Future research should address these limitations through:

- Integration of dynamic stability indices.
- Validation using real-world PMU data with measurement noise.
- Extension to networks with high distributed generation penetration.
- Development of uncertainty quantification methods for ML predictions.

The findings demonstrate that ensemble machine learning methods, particularly GB and RF, provide reliable and computationally efficient solutions for voltage stability assessment in modern distribution networks. The proposed methodology offers a practical framework for enhancing grid resilience through proactive stability monitoring and control.

## Conclusion

The research presented the predictive performance of machine learning algorithms in assessing voltage instability within electrical distribution networks. By proposing the FVSI as a predictive tool, we successfully demonstrated the potential for rapid identification of voltage instability, a crucial factor in preventing outages and ensuring operational reliability. The findings from the case studies on both 35-bus and 53-bus networks reinforce the capability of ML techniques, particularly Random Forest and Gradient Boosting, to deliver accurate predictions swiftly. The results demonstrate that the Gradient Boosting model delivered outstanding accuracy, achieving RMSE values of just 0.0002 for the 53-bus system and 2.419e-05 for the 35-bus system. Random Forest also performed strongly, with corresponding RMSE values of 0.0039 and 0.00120. These outcomes confirm the models’ effectiveness for real-time voltage stability assessment. This paper contributes significantly to the field by offering a practical solution that caters to the constraints of modern power systems, emphasizing the necessity for real-time monitoring and quick decision-making in voltage stability management.

This paper not only addresses existing challenges but also lays the groundwork for future advancements in power system resilience and reliability. So Future work will focus on enhancing the robustness of the proposed models under high DG penetration and integrating additional predictive features to improve instability forecasting in complex grid scenarios. While this study utilizes FBLFA to generate supplementary data such as voltage profiles at various buses within selected feeders from Bahir Dar distribution network in Ethiopia and incorporates essential line and load data sourced from the Ethiopian Electric Utility (EEU), we recognize a key limitation as the absence of field measurement data from PMUs or SCADA systems. This omission poses a significant challenge, as it affects the model’s ability to generalize effectively to noisy or incomplete real-time data, which is critical for practical deployment.

## Data Availability

The datasets used and/or analyzed during the current study are available from the corresponding author on reasonable request.
